# Prevention of neuronal apoptosis by astrocytes through thiol-mediated stress response modulation and accelerated recovery from proteotoxic stress

**DOI:** 10.1038/s41418-018-0229-x

**Published:** 2018-11-02

**Authors:** Simon Gutbier, Anna-Sophie Spreng, Johannes Delp, Stefan Schildknecht, Christiaan Karreman, Ilinca Suciu, Thomas Brunner, Marcus Groettrup, Marcel Leist

**Affiliations:** 1 In vitro Toxicology and Biomedicine, Dept inaugurated by the Doerenkamp-Zbinden foundation, University of Konstanz, 78457 Konstanz, Germany; 2 Research Training Group RTG1331, University of Konstanz, Konstanz, Germany; 3 Konstanz Research School Chemical Biology, University of Konstanz, Constance, Germany; 4 Cooperative Doctorate College InViTe, University of Konstanz, Konstanz, Germany; 5 Biochemical Pharmacology, Department of Biology, University of Konstanz, Konstanz, Germany; 6 Division of Immunology, Department of Biology, University of Konstanz, D-78457 Konstanz, Germany; 7 CAAT-Europe, University of Konstanz, 78457 Konstanz, Germany

## Abstract

The development of drugs directly interfering with neurodegeneration has proven to be astonishingly difficult. Alternative therapeutic approaches could result from a better understanding of the supportive function of glial cells for stressed neurons. Therefore, here, we investigated the mechanisms involved in the endogenous neuro-defensive activity of astrocytes. A well-established model of postmitotic human dopaminergic neurons (LUHMES cells) was used in the absence ('LUHMES' mono-culture) or presence ('co-culture') of astrocytes. Inhibition of the LUHMES proteasome led to proteotoxic (protein aggregates; ATF-4 induction) and oxidative (GSH-depletion; NRF-2 induction) stress, followed by neuronal apoptosis. The presence of astrocytes attenuated the neuronal stress response, and drastically reduced neurodegeneration. A similar difference between LUHMES mono- and co-cultures was observed, when proteotoxic and oxidative stress was triggered indirectly by inhibitors of mitochondrial function (rotenone, MPP^+^). Human and murine astrocytes continuously released glutathione (GSH) into the medium, and transfer of glia-conditioned medium was sufficient to rescue LUHMES, unless it was depleted for GSH. Also, direct addition of GSH to LUHMES rescued the neurons from inhibition of the proteasome. Both astrocytes and GSH blunted the neuronal ATF-4 response and similarly upregulated NRF-1/NFE2L1, a transcription factor counter-regulating neuronal proteotoxic stress. Astrocyte co-culture also helped to recover the neurons’ ability to degrade aggregated poly-ubiquitinated proteins. Overexpression of NRF-1 attenuated the toxicity of proteasome inhibition, while knockdown increased toxicity. Thus, astrocytic thiol supply increased neuronal resilience to various proteotoxic stressors by simultaneously attenuating cell death-related stress responses, and enhancing the recovery from proteotoxic stress through upregulation of NRF-1.

## Introduction

Neuronal stress response signals are a critical element in the pathogenesis of various neurodegenerative diseases. Endogenous mechanisms of neuronal resilience to stress are thus of high interest to develop new strategies for the modulation of neurodegenerative diseases, like Parkinson’s disease (PD). The main hallmark of PD is the degeneration of dopaminergic neurons in the *substantia nigra*. The pathogenic mechanisms thought to take an important role in this process comprise mitochondrial dysfunction with ensuing oxidative stress, impairment of protein turnover, evidenced by dysfunction of the ubiquitin proteasome system (UPS) and aggregation of proteins like α-synuclein [1–3].

To study neurodegeneration and neuroprotection in PD, usually pathological features observed in affected human brains are modelled. For instance, impairment of mitochondrial function is triggered by their genetic inactivation [4, 5] or by the toxicants 1-methyl-4-phenylpyridinium (MPP^+^) and rotenone [6, 7]. Proteotoxic stress can be induced by overexpression or injection of α-synuclein, or by direct inactivation of the UPS by proteasome inhibitors [8]. The latter triggers several PD-relevant processes, such as aggregation of α-synuclein and the death of dopaminergic neurons [9–11]. One of the cellular stress responses induced by proteasome inhibition is the upregulation of NRF-1 (=TCF-11/NFE2L1; not to be confused with the mitochondrial biogenesis factor NRF1), a transcription factor involved in the regulation of proteasome synthesis [12].

The different pathological processes linked to PD are highly interconnected, and there are multiple examples for impairment of the UPS occurring because of mitochondrial impairment [2, 8] and vice versa [13]. For instance, the proteasome biogenesis factor NRF-1 has been associated with increased cellular resistance to mitochondrial impairment [14].

Using a multi-omics approach, we recently identified the proteotoxic stress-related transcription factor (TF) ATF-4 as coordinator of the neuronal stress response following mitochondrial respiratory chain inhibition [15]. Regulation of ATF-4 may indicate a cellular demand for thiol supply, as it upregulates cystine import and cysteine generation via the transsulfuration pathway [15]. Interestingly, shortage of cysteine has also been identified as a critical factor determining cell death after proteasome inhibition [16].

We also observed that co-culturing neurons with astrocytes extends neuronal survival and provides trophic support under otherwise unfavourable conditions [17, 18]. Astrocytes protected neurons also against nitric oxide [19]. This is in line with many studies describing or implying astrocytic neuroprotection in vitro and in vivo [20–24]. A better understanding of how astrocytes protect neurons, and how their presence affects neuronal stress responses might result in new strategies to treat neurodegenerative diseases.

Therefore, here, we studied the mechanisms of astrocytic neuroprotection. As a model system, we used human neurons generated from the LUHMES cell line. Such cells are known to differentiate to fully postmitotic and electrically active neurons with high expression of dopaminergic features, such as the dopamine transporter [25]. These neurons were exposed to mitochondrial toxicants (MPP^+^ and rotenone) or proteasome inhibitors. To provide a homogeneous (> 99% pure) and defined astrocytic population, we differentiated neural stem cells to glia. These ‘murine astrocytes generated from embryonic stem cells’ (mAGES) [17, 26, 27] displayed all typical metabolic features of tissue astrocytes. By comparing the responses of neurons cultured alone ('LUHMES') or with astrocytes ('co-cultures'), pronounced neuroprotection by glia was observed. Astrocyte-derived GSH was identified as a key factor that not only attenuates neuronal stress (ATF-4) responses, but also increased neuronal resilience by supporting the upregulation of NRF-1.

## Results

### Different sensitivities of neurons in mono- and co-culture

**Fig. 1:**
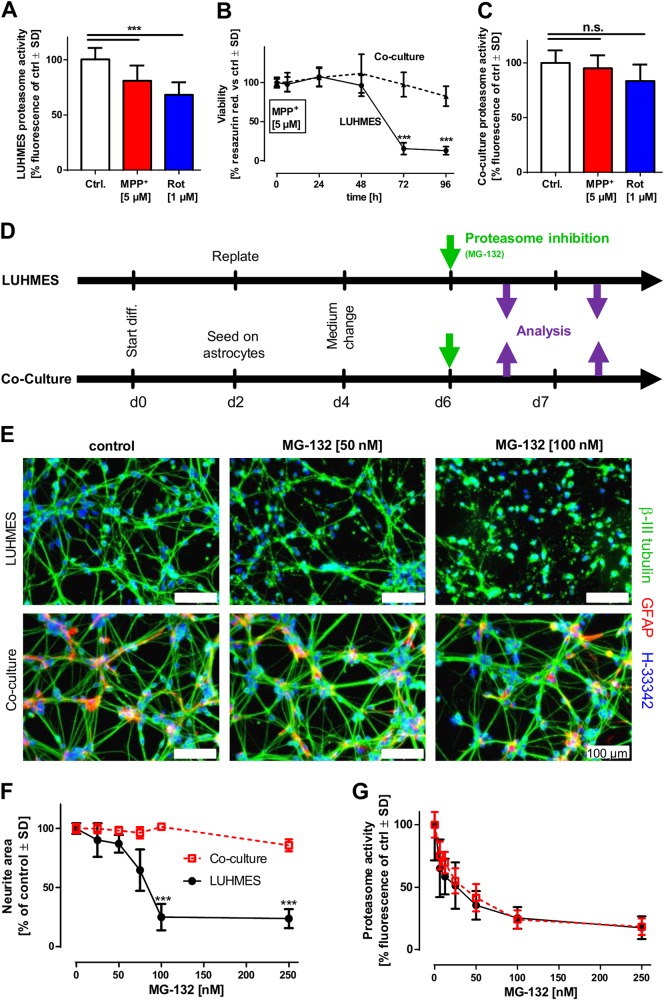
Increased tolerance to neuronal proteasome inhibition in the presence of astrocytes. **a** LUHMES cells (d6) were treated with the indicated concentrations of MPP^+^ or rotenone for 24 h. Proteasome activity was assessed fluorometrically. **b** LUHMES cells (d6) were treated with MPP^+^ [5 µM] either in mono- or in co-culture with astrocytes for the indicated time periods. Viability was assessed by measuring resazurin reduction at the indicated time points. **c** LUHMES astrocyte co-cultures were treated with MPP^+^ [5 µM] or rotenone [1 µM] for 24 h. Proteasome activity was assessed fluorometrically. **d** LUHMES cells in mono- and co-culture with astrocytes were differentiated according to the depicted differentiation scheme. LUHMES cells were replated at d-1, and differentiation started by replacing the proliferation medium (PM) with differentiation medium (DM) containing tetracycline, cAMP and GDNF on d0. After 2 days (d2), LUHMES cells were re-plated either as mono-cultures or seeded on top of pre-differentiated astrocytes (mAGES). The medium was exchanged on d4 and 'mature cells' were ready on d6 for toxicant exposure. **e** LUHMES cells (d6) mono- or co-cultures were exposed to the indicated concentrations of MG-132 for 24 h. Differential toxicity was assessed by immunocytochemistry staining with antibodies against β-III tubulin and GFAP. Nuclei were counterstained with H-33342. **f** Toxicity of MG-132 on LUHMES mono- and co-cultures was assessed by measuring the neurite integrity after cells were exposed for 24 h to MG-132 at the indicated concentrations. **g** Proteasomal inhibition by MG-132 in LUHMES mono- and co-cultures was assessed by measuring proteasome activity fluorometrically. For A-F, differences were tested for significance by one-way ANOVA, followed by Dunnett’s post hoc test, n.s.: non-significant, *: *p* < 0.05, **: *p* < 0.01, ***: *p* < 0.001 for comparison of treatments to the respective untreated controls. Data are means ± SD of three independent experiments

As mitochondrial impairment and proteasomal dysfunction are tightly interconnected in the pathogenesis of PD, we investigated whether such an interdependence is also observed in the model system used for our study. We observed that both rotenone and MPP^+^ decreased proteasome activity of LUHMES dopaminergic neurons (Fig. 1a). Moreover, the two compounds led to the accumulation of ubiquitinated proteins (Fig. S1A+B) and to the formation of intracellular protein aggregates (Fig. S2A+B). Having established these measures of proteotoxic stress, we asked how the presence of astrocytes (mAGES) would affect the neuronal response: we exposed LUHMES neurons to MPP^+^ in the presence or absence of astrocytes, and noticed that the presence of even few astrocytes (10% of the neuronal cell number) was sufficient to promote a very robust neuroprotection (Fig. 1b). We also observed that the impairment of the UPS was less pronounced in co-cultures than in LUHMES mono-cultures (Fig. 1c). Notably, a large series of control experiments (data not shown) indicated that the astrocytes did not alter neuronal exposure to the toxicant (e.g. MPP^+^ uptake).

While these introductory experiments indicated a potential role of astrocytes in attenuating neuronal proteotoxic stress, further studies required a sharper tool for proteasome inhibition (MPP^+^ affects proteasome activity, neuronal energy generation and ROS formation). To obtain clear mechanistic data from LUHMES mono-cultures vs astrocyte co-cultures (Fig. 1d), we modulated the proteasome activity directly. LUHMES neurons were found to be extremely sensitive to cell death triggered by the proteasome inhibitor MG-132 (50 nM) (Fig. 1e, f). This cell death was preceded by a strong accumulation of ubiquitinated proteins and protein aggregates within the neurons (Fig. S1C+D and Fig. S4A+B). In co-cultures, neurons were completely protected by astrocytes at drug concentrations of up to 250 nM (Fig. 1e, f), while the degree of proteasomal inhibition in LUHMES was not affected by the astrocytes (Fig. 1g). Notably, neuronal protection from MG-132 was also observed with human astrocytes (Fig. S3).

### Stress response in neurons following proteasome inhibition

**Fig. 2:**
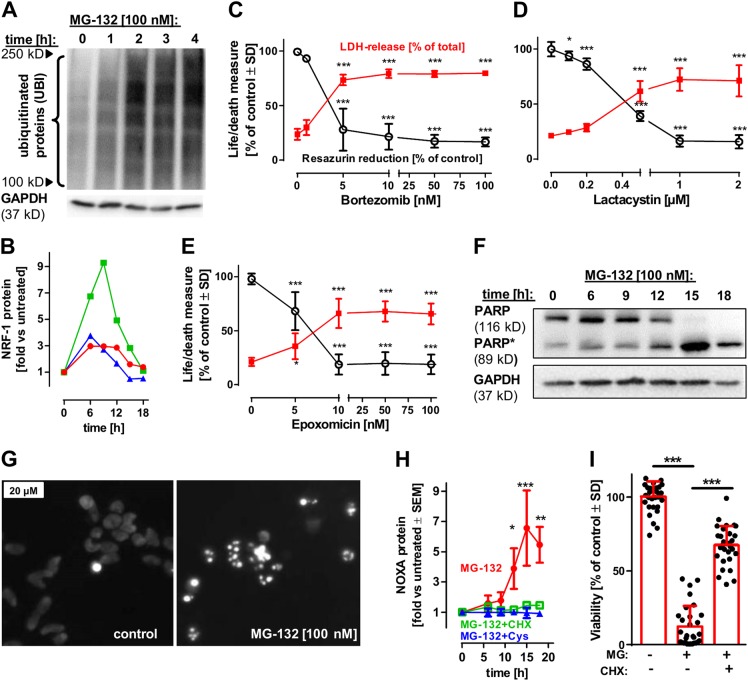
Formation of protein aggregates and triggering of neuronal apoptosis. **a** LUHMES cells (d6) were treated with MG-132 [100 nM] for the indicated time periods. After incubation, cells were lysed and analysed by western blot using anti-ubiquitin and anti-GAPDH antibodies. One of two similar data sets is shown. **b** LUHMES cells (d6) were treated with MG-132 [100 nM] for the indicated time periods. Then, the cells were lysed and analysed by western blot with anti-NRF-1 and anti-GAPDH antibodies. The ratios of NRF-1/GAPDH were quantified densitometrically and normalised to untreated controls (displayed as NRF-1 protein). The lines in red, blue and green show the NNRF-1 levels of three independent experiments. Standard statistics were not displayed, as comparison to the control (without SD) would exaggerate apparent significances, and as the time-series data points are not independent of one another. Use of a repeated connected measures ANOVA with Dunnett’s post hoc test indicates *p* = 0.02; alternatively, when the values of 6 and 9 h (pooled) were compared to 0 h, by a standard one-sample *t* test, *p* was 0.019. **c–e** Cell death of LUHMES cells following proteasome inhibition by bortezomib, clasto-lactacystin β-lactone (lactacystin) and epoxomicin was monitored. Cells were exposed to the indicated concentrations of the compounds for 24 h. Viability was assessed measuring resazurin reduction and LDH release. Differences were tested for significance by one-way ANOVA, followed by Dunnett’s post hoc test, *: *p* < 0.05, ***: *p* < 0.001 for comparison of treatments to untreated control. Data are means ± SD of three independent experiments. **f** LUHMES cells (d6) were treated with MG-132 [100 nM] for the indicated time periods. After incubation, cells were lysed and analysed by western blot, using anti-PARP and anti-GAPDH antibodies. One of three similar experiments is displayed. **g** LUHMES cells (d6) were treated with MG-132 [100 nM]; then the nuclear morphology and DNA condensation were visualised by using the DNA intercalating dye H-33342. **h** LUHMES cells (d6) were treated with MG-132 [100 nM] in the presence or absence of cycloheximide (CHX) [10 µM] or cysteine (Cys) [1 mM] for the indicated time periods. After incubation, cells were lysed and analysed by western blot using anti-NOXA and anti-GAPDH antibodies. Induction of NOXA was quantified densitometrically. Differences were tested for significance by two-way ANOVA (treatment × time), followed by Tukey’s post hoc test, *: *p* < 0.05, **: *p* < 0.01, ***: *p* < 0.001 for the comparison of MG-132 treatment at the given time points to combined treatment with MG-132 with either CHX or Cys. Data are means ± SEM of three independent experiments. **i** The effect of cycloheximide [10 µM] on neuronal survival following proteasome inhibition was investigated by measuring neurite integrity as a surrogate for viability. Differences were tested for significance by one-way ANOVA, followed by Bonferroni’s post hoc test, ***: *p* < 0.05 for multiple comparisons. Bars show means ± SD of three independent experiments; black dots show values of all technical replicates run within these experiments

We further characterised the different responses of neurons in the presence or absence of astrocytes. Accumulation of poly-ubiquitinated proteins was detectable as soon as 2 h after MG-132 exposure (Fig. 2a). This was followed by an upregulation of key TF associated with proteotoxic stress, ATF-4 and NRF-1 [12, 28, 29] after 6 h (Fig. 2b, Fig. S4A). In order to investigate whether the relevant target of MG-132 in neurons was the proteasome, we tested the effect of three other proteasome inhibitors. LUHMES were found to be highly sensitive also to bortezomib, lactacystin and epoxomicin (Fig. 2c–e). As the proteasome is the common target of these highly diverse inhibitors (at low concentrations), we conclude that the neuronal cell death observed is triggered by reduced proteasome activity.

We observed typical apoptotic features after prolonged (>12 h) proteasome inhibition: PARP and fodrin cleavage (Fig. 2f, Fig. S5C), nuclear fragmentation (Fig. 2g) and caspase-3 activation. Cell death and caspase-3 processing were blocked by caspase inhibitors (Fig. S5A+B), while the accumulation of ubiquitinated proteins persisted in caspase-inhibited cells (Fig. S5D).

As a further sign of the induction of the mitochondrial apoptosis pathway, we observed an increased expression of the pro-apoptotic protein NOXA prior to caspase activation (starting 12 h after exposure to MG-132; Fig. 2h, Fig. S6A). Inhibition of protein synthesis by cycloheximide prevented NOXA induction and rescued neurons from cell death (Fig. 2i, Fig. S6A). The finding that cycloheximide was still neuroprotective, when added up to 10 h after MG-132 (Fig. S6B), suggests that the biosynthesis of pro-apoptotic proteins (e.g. NOXA) is initiated at 10–12 h after proteasomal impairment, and that the shifted balance of pro-/anti-apototic proteins eventually leads to caspase activation and apoptosis induction.

Concerning cell death quantification, it is important that neurites and cell bodies (somata) may undergo different death programmes. For neuroprotection experiments, it was therefore important to verify that not only neuronal somata survived, but also the neurite structure remained intact [30]. We confirmed that this was the case for neuronal protection by astrocytes (Fig. 1e) or by the caspase inhibitor zVADfmk (Fig. S4B). For all further experiments, the intactness of the neurites was considered as the most stringent measure of neuroprotection.

### Rescue of neurons by cysteine

**Fig. 3:**
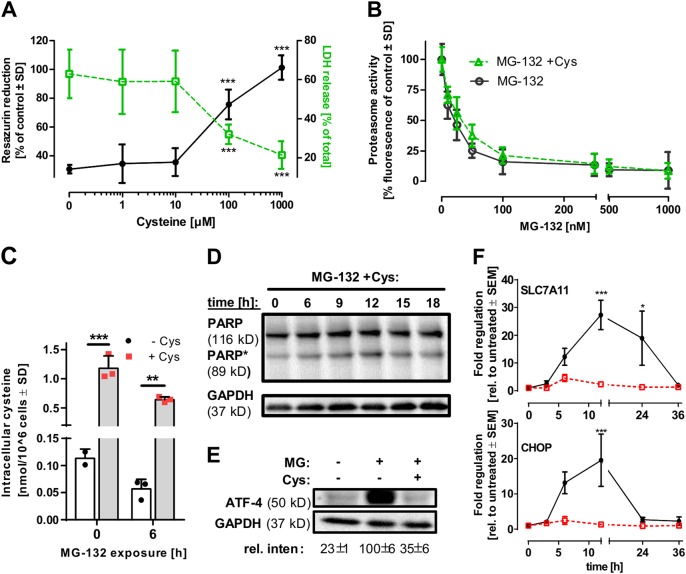
Protection by external cysteine from proteotoxic neuronal stress. **a** LUHMES cells (d6) were treated with MG-132 [100 nM] and the indicated concentrations of L-cysteine for 24 h. Viability was assessed by measuring resazurin reduction and LDH release. Differences were tested for significance by one-way ANOVA followed by Dunnett’s post hoc test, ***: *p* < 0.001. Data are means ± SD of three independent experiments. **b** Proteasomal inhibition by MG-132 in the presence or absence of 1 mM L-cysteine was assessed in LUHMES cells by measuring proteasome activity fluorometrically. Data are means ± SD of three independent experiments. **c** Intracellular cysteine levels of LUHMES cells exposed to MG-132 [100 nM] for 0 or 6 h in the presence or absence of 1 mM extracellular L-cysteine were measured by amino acid analysis. Data are means ± SD of three independent experiments. **d** LUHMES cells (d6) were treated with MG-132 [100 nM] and L-cysteine [1 mM] for the indicated time periods. After incubation, cells were lysed and analysed by western blot using anti-PARP and anti-GAPDH antibodies (data representative of three experiments). **e** LUHMES cells (d6) were treated with MG-132 [100 nM] in the presence or absence of 1 mM L-cysteine for 6 h. After incubation, cells were lysed and analysed by western blot using anti-ATF-4 and anti-GAPDH antibodies. ATF-4 induction was quantified densitometrically. Numbers indicate band intensities (means ± SD of three independent experiments, normalized to GAPDH) relative to MG-132-treated samples. **f** Differentiated d6 LUHMES cells were exposed to MG-132 [100 nM] in the presence or absence of 1 mM L-cysteine. Changes in mRNA levels of the ‘cystine/glutamate transporter (SLC7A11)’ and the ‘DNA damage inducible transcript 3 (CHOP)’ were quantified after the indicated time periods by qPCR. Data are means ± SEM of three independent experiments. Differences in **c** and **f** were tested for significance by two-way ANOVA (treatment × time), followed by a Bonferroni post hoc test, *: *p* < 0.05, **: *p* < 0.01, ***: *p* < 0.001 for comparison amongst treatments at the given time points

When evaluating the literature for mediators of astrocytic protection, we realised that neurons rely heavily on glia to maintain their cysteine pool, and this dependence may get more pronounced in the presence of MG-132 (known to deplete cellular cysteine levels) [16]. We found cysteine supplementation to reduce neuronal sensitivity to MG-132 (Fig. 3a), lactacystin and bortezomib (Fig. S7). While proteasome inhibition was not altered by cysteine (Fig. 3b), the very low intracellular cysteine levels of neurons increased about 10-fold (Fig. 3c). Caspase activation and induction of apoptosis, as monitored by PARP cleavage (Fig. 3d) and NOXA levels (Fig. 2h), were completely prevented in the presence of cysteine. Moreover, the activation of ATF-4 and the induction of its target genes (CHOP and the cysteine transporter SLC7A11) was blocked (Fig. 3e, f and Fig. S8), indicating a pronounced attenuation of the neuronal stress response.

### Rescue of neurons by external GSH

**Fig. 4:**
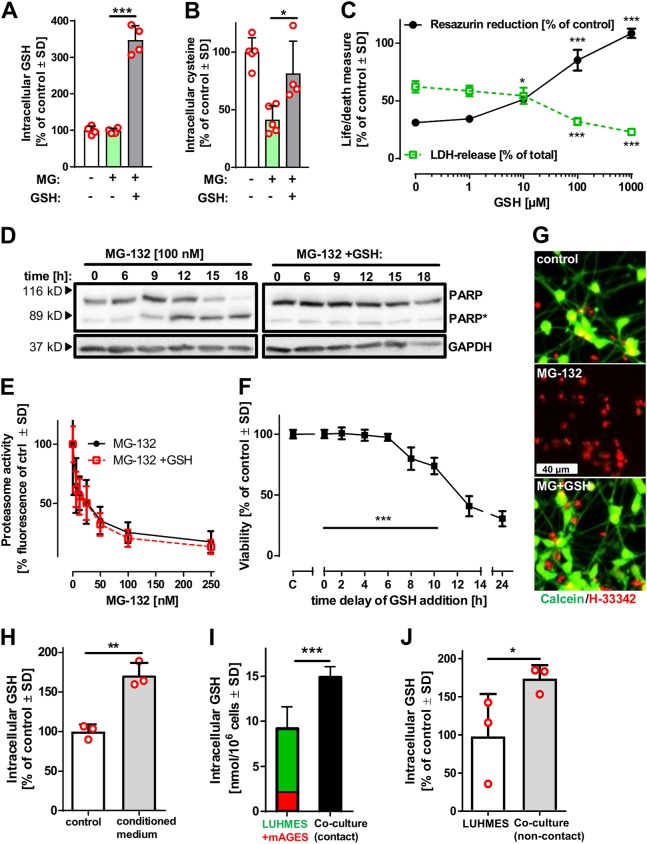
Rescue of neurons from proteasome inhibition by glutathione supply. **a**, **b** Intracellular GSH and cysteine levels of LUHMES cells that were exposed to MG-132 [100 nM] for 6 h in the presence or absence of extracellular GSH [1 mM] were measured by amino acid analysis. Differences were tested for significance by one-way ANOVA followed by Bonferroni’s post hoc test, *: *p* < 0.05, ***: *p* < 0.001 for comparison of all bars. Data are from ≥ four experiments (individual data shown as red circles). **c** LUHMES (d6) cells were incubated with MG-132 [100 nM] and the indicated concentrations of GSH for 24 h. Viability was assessed by measuring resazurin reduction and LDH release. Differences were tested for significance by one-way ANOVA followed by Dunnett’s post hoc test, *p < 0.05, ***p < 0.001 for comparison of GSH supplementation to MG-132 single treatment (three independent experiments). **d** LUHMES (d6) cells were treated with MG-132 [100 nM] and GSH [1 mM] for the indicated time periods. After incubation, cells were lysed and analysed by western blot using anti-PARP and anti-GAPDH antibodies. Representative blots (*N* = 3 experiments) are shown. PARP*: apoptotically cleaved PARP. **e** Proteasomal inhibition with the indicated concentrations of MG-132 in the presence or absence of GSH [1 mM] was assessed in LUHMES (d6) cells by measuring the proteasome activity fluorometrically. Data are means ± SD of four independent experiments. **f** LUHMES cells (d6) were treated with MG-132 [100 nM], and GSH [1 mM] was added at various indicated time points after the start of MG-132 exposure. Viability was assessed using calcein-AM/H-33342 staining at 24 h after the start of the MG-132 exposure. Double-positive cells were counted by automated microscopy and normalised for all H-33342-positive cells. Differences were tested for significance (*N* = 3 experiments) by one-way ANOVA followed by Dunnett’s post hoc test, ***: *p* < 0.001 for comparison of samples with GSH added vs MG-132 treatment without GSH (= 24 h data point). **g** Cells were treated as described in (**f**) and stained after 24 h of MG-132 treatment with the vital dye calcein-AM and H-33342. Representative pictures for control, MG-132 [100 nM] and cells treated with MG-132 [100 nM] *plus* GSH (with a time delay of 8 h). **h** Intracellular GSH levels of cells incubated for 6 h either with standard differentiation medium or astrocyte-conditioned medium were determined by amino acid analysis. Differences were tested for significance by Student’s *t* test (three independent experiments, indicated as red circles) to compare conditioned medium with standard medium control. **i** Combined GSH levels of LUHMES (d6) and mAGES mono-cultures, as well as GSH levels of ‘direct-contact co-cultures’. Values were normalised to cell number. Student’s *t* test: ***: *p* < 0.001 for comparison of total GSH content in co-cultures with the sum of GSH contents of the mono-cultures. Data are means ± SD of three independent experiments. **j** GSH levels of LUHMES cells cultured on a cover slip positioned 1 mm above mAGES (non-contact co-cultures) and LUHMES cells cultured alone were measured. *: *p* < 0.05 according to Student’s *t* test (three independent experiments, paired samples)

Cysteine itself is unlikely to be released from astrocytes, as this amino acid has a high excitotoxic potential [31, 32]. However, the cysteine-containing tripeptide GSH is a well-established candidate metabolite transferred from glia to neurons [33–36]. Therefore, we investigated the effect of GSH on neuronal survival after MG-132 exposure. Addition of GSH to neuronal mono-cultures leads to an intracellular GSH increase (Fig. 4a), and blocked cysteine depletion after proteasome inhibition by MG-132 (Fig. 4b). Furthermore, GSH blocked MG-132-induced cell death (Fig. 4c) and prevented apoptotic PARP cleavage (Fig. 4d), while the inhibition of the proteasome by MG-132 was not altered by GSH (Fig. 4e). Importantly, GSH was still effective at preventing cell death when added 8–10 h after MG-132 (Fig. 4f, g). To get evidence on the role of astrocytes in providing GSH, we incubated neurons with astrocyte-conditioned medium, and observed an increase in neuronal GSH under this condition (Fig. 4h). The increase in neuronal GSH levels triggered by increasing the fractions of glia-conditioned medium (from mAGES and human astrocytes) was paralleled by an increased survival after MG-132 exposure (Fig. S9A-C) and scavenging of thiols in conditioned medium from astrocytes blunted the protective properties (Fig. S9D+E). In the next step, we compared the GSH content of co-cultures (90% neurons) with the content of neurons alone or with separate neurons and astrocytes. Co-cultures had significantly higher GSH levels than the two separate mono-cultures combined (Fig. 4i). Moreover, LUHMES cultured on coverslips 1 mm above mAGES and then analysed separately had significantly higher GSH levels compared to LUHMES cultured in the absence of mAGES (Fig. 4j). We conclude from this that the neuronal GSH was increased in the presence of astrocytes, similar as observed with conditioned medium.

### Alterations in the neuronal stress response by GSH

**Fig. 5:**
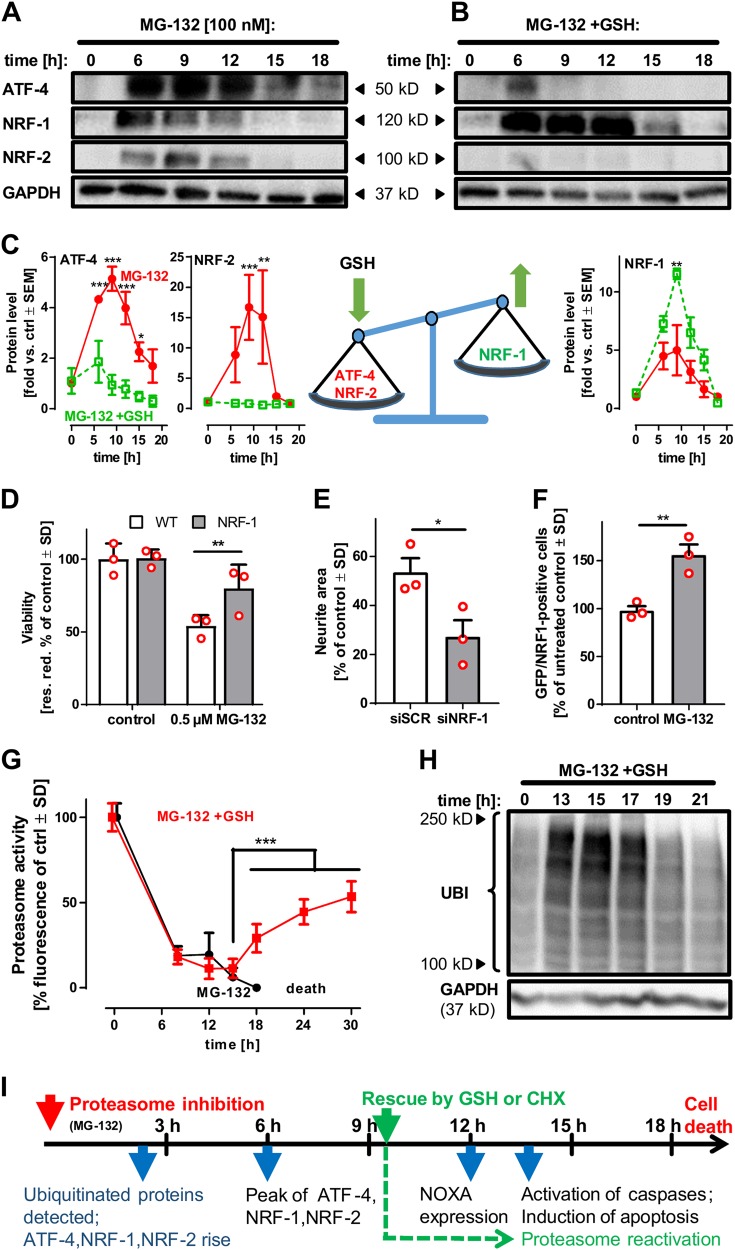
Influence of external thiols on the balance between ATF-4, NRF-1 and NRF-2. **a**, **b** To address the differences in the neuronal stress response following proteasome inhibition in the absence (**a**) or presence (**b**) of GSH [1 mM], cells were treated with MG-132 [100 nM] for the indicated time periods. After incubation, cells were lysed and analysed by western blot using anti-ATF-4, anti-NRF-1, anti-NRF-2 and anti-GAPDH antibodies. **c** Densitometric quantification of A and B and a schematic depiction of the influence of GSH on the stress response following MG-132 exposure. Differences were tested for significance by two-way ANOVA (treatment × time), followed by a Bonferroni post hoc test, *: *p* < 0.05, **: *p* < 0.01, ***: *p* < 0.001 for comparison amongst treatments at the given time points. Data are fold change vs control ± SEM of three independent experiments. **d** HEK-293 cells over-expressing NRF-1 and wild-type (WT) cells were incubated for 48 h with MG-132 [0.5 µM]. Viability was assessed by measuring resazurin reduction. Differences were tested for significance by two-way ANOVA (treatment × genotype), followed by a Bonferroni post hoc test, **: *p* < 0.01. Individual data of the three independent experiments are shown as red circles. **e** LUHMES cells (d2) were transfected with siRNA against NRF-1 or scrambled siRNA. On day 6 of differentiation, cells were incubated with MG-132 [100 nM], and then GSH [1 mM] was added with a time delay of 7 h. Viability was measured after 24 h, using the vital dye calcein-AM and the DNA stain H-33342. The neurite area of the cell cultures was assessed by automated microscopy. *: *p* < 0.05 (*t* test). **f** LUHMES cells (d2) were transfected with a plasmid driving the expression of NRF-1 and GFP. On d6, cells were treated with MG-132 [100 nM] for 18 h. The viability was assessed by calcein-AM/ H-33342 staining. Double-positive cells were counted by automated microscopy. **: *p* < 0.01 (*t* test, with individual data points shown as red circles). **g** Proteasomal recovery after exposure to MG-132 [100 nM] in the presence or absence of 1 mM GSH was assessed in LUHMES cells (d6) by measuring proteasome activity fluorometrically after the indicated incubation times. At 24 h after exposure to MG-132, proteasomal activity became undetectable in cells treated with MG-132 only (due to the death of the cells). Differences were tested for significance by two-way ANOVA (time × GSH treatment), followed by Dunnett’s post hoc test, ***:*p* < 0.001 for comparison of the GSH-treated cells at 18, 24 and 30 h (recovery phase) vs the 15-h time point (maximum inhibition as the baseline for recovery). **h** Cells were treated with MG-132 [100 nM] for the indicated time periods in the presence of GSH [1 mM]. After incubation, cells were lysed and analysed by western blot using anti-ubiquitin (UBI) and anti-GAPDH antibodies (an individual experiment in confirmation of fluorescence data). **i** Time line of events in neurons following MG-132 exposure. The red arrow indicates the start of MG-132 exposure. The green arrow shows the latest time point for complete rescue by GSH and CHX. The blue arrows indicate events associated with the stress response and cell death

To further characterise the effect of GSH supplementation on the neuronal stress response and cell death, we monitored the protein levels of the stress-associated TF ATF-4, NRF-2 and NRF-1 (Fig. 5a–c). In cells treated with MG-132 only, these TF were upregulated from 6 h until 12 h after MG-132 exposure (Fig. 5a, c). Cells co-treated with GSH displayed a weak ATF-4 and no detectable NRF-2 signal, while NRF-1 levels were elevated (Fig. 5b, c). Thus, GSH modulated different stress response pathways in opposite ways. In line with this observation, the upregulation of ATF-4 target genes was attenuated in the presence of GSH, while NRF-1 target genes showed an increased transcription (Fig. S10A+B). As NRF-2 is predominantly an indicator of oxidative stress, its downregulation by GSH confirms that proteasome inhibition triggers neuronal stress, which is blunted by an improved GSH supply.

The role of ATF-4 in neurodegeneration is ambiguous [15]. This TF, when triggered by ER stress, can take a pro-apoptotic role, e.g. by induction of the apoptosis enhancer DDIT-4 [37, 38]. We observed here that GSH blunted the DDIT-4 response (Fig. S11A). Moreover, partial knockdown of DDIT-4 in LUHMES attenuated cell death triggered by MG-132, while the counter-regulatory NRF-1 response was enhanced (Fig. S11B–D). Thus, attenuation of the ATF-4 arm of the stress response may contribute to neuroprotection or prolong the period of neuronal resilience. Notably, ATF-4 induced by amino acid starvation, also regulates pro-survival pathways (e.g. cystine import and transsulfuration) [15, 39]. The observed downregulation by GSH would also be in line with this role, as GSH counteracted the cysteine deficit (Fig. 4b), and abolished the need for increased GSH synthesis. Since all attempts to deplete LUHMES of ATF-4 failed, we produced ATF-4-deficient HEK-293 by Crispr/Cas technology. Such ATF-4 knockout cells failed to regulate typical ATF-4 target genes following exposure to MG-132 (Fig. S12A+B), and they had a slightly increased sensitivity to proteasome inhibition (Fig. S14C). This suggests that downregulation of ATF-4 alone may not be sufficient to protect cells from apoptosis and to explain the neuroprotective effect of GSH. The parallel upregulation of NRF-1 may offer such an explanation. NRF-1 may provide neuroprotection by upregulating the proteasome [12, 28, 29] and thereby attenuating proteotoxic stress. As a proof of principle, we overexpressed NRF-1 in HEK-293 cells and exposed them to MG-132. Under this condition, we observed a significant increase in survival (Fig. 5d–f), while knocking out NRF-1 increased the cell susceptibility and reduced the window in which cells could be rescued by cysteine (Fig. S15). In LUHMES, attenuation of the NRF-1 response (by siRNA) reduced the protection provided by GSH (Fig. 5e; Fig. S14A), while overexpression of NRF-1 increased the neuronal survival following proteasome inhibition (Fig. 5f; Fig. S14C).

To obtain some evidence on the mechanisms responsible for neuronal protection by NRF-1 superinduction, we studied proteasome activity and protein aggregation over time. Indeed, we observed that the addition of GSH allowed cells to recover proteasome activity (Fig. 5g). The return of enzymatic activity was followed by a clearance of ubiquitinated proteins to baseline levels after 24 h (Fig. 5h). In line with this, GSH supplementation significantly reduced intracellular protein aggregation following MG-132 exposure (Fig. S13A+B).

These findings suggest the following time course of events (Fig. 5i): stress response factors are induced in parallel with the accumulation of ubiquitinated proteins, starting few hours after addition of MG-132. After about 10 h, neurons reach the point-of-no-return and start succumbing to proteotoxic stress. Death is executed by the activation of caspases at 12–15 h. GSH is likely to have a dual effect, first by attenuating oxidative stress (e.g. attenuated induction of DDIT-4) to such an extent that the point-of-no-return is delayed by some hours. We suggest that the second effect is enhanced recovery of proteasome activity and cell function related to upregulated NRF-1.

### Astrocytic modulation of neuronal stress responses and neuroprotection

**Fig. 6:**
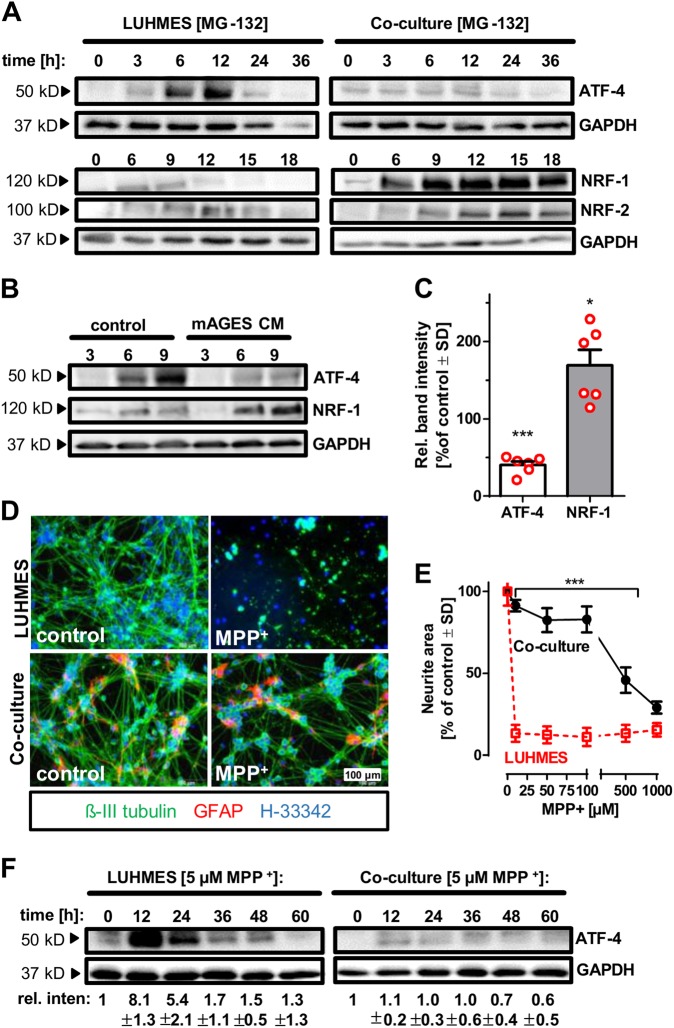
Alterations in neuronal stress response and rescue by astrocytes. **a** LUHMES cells (d6) and LUHMES astrocyte co-cultures were treated with MG-132 [100 nM] for the indicated time periods. After incubation, cells were lysed and analysed by western blot using anti-ATF-4, anti-NRF-1, anti-NRF-2 and anti-GAPDH antibodies (one experiment, representative of two, is shown). **b**, **c** LUHMES cells (d6) were incubated with either standard differentiation medium (control) or astrocyte-conditioned medium (mAGES CM), and treated with MG-132 [100 nM] for the indicated time periods. After incubation, cells were lysed and analysed by western blot using anti-ATF-4, anti-NRF-1 and anti-GAPDH antibodies. After densitometric analysis (normalised for GAPDH), the 6  and 9 h bands (pooled data) of NRF-1 and ATF-4 were compared between the media conditions. Differences between normal and conditioned medium (normal set to 100%) are shown (*n* = 3). Means, error bars, as well as individual data (red circles) are displayed, *: *p* < 0.05 (Student’s *t* test, Benjamini-Hochberg-corrected; conditioned vs control medium). **d**, **e** Neuronal survival of co- and mono-cultured LUHMES was assessed 72 h after incubation with the indicated concentrations of MPP^+^. Representative pictures of cells treated with MPP^+^ [5 µM] are shown in (**d**) (note: astrocytes indicated in red). Quantification of the neurite area using automated microscopy is shown in (**e**). Differences were tested for significance by two-way ANOVA, followed by a Tukey post hoc test, ***: *p* < 0.001 for comparison of co-cultures vs mono-cultures at given MPP^+^ doses. Data are means ± SD of three independent experiments. **f** LUHMES cells (d6) and co-cultures were treated with MPP^+^ [5 µM] for the indicated time periods. After incubation, cells were lysed and analysed by western blot using anti-ATF-4 and anti-GAPDH antibodies. The band intensities from three such experiments were quantified (ATF-4/GAPDH ratios), and the relative band intensities (0 h = 1) are displayed as means ± SD

Having established the dominant signalling events associated with GSH-mediated protection of neurons, we now asked whether astrocytes would lead to a similar modification of the stress response network. Indeed, the presence of astrocytes suppressed the activation of ATF-4 and increased the NRF-1 response in neurons (Fig. 6a, Fig. S16). When LUHMES were incubated with astrocyte-conditioned medium and treated with MG-132, the ATF-4 response was also weaker than in cells cultured in normal differentiation medium, while the NRF-1 response was increased (Fig. 6b, c). This indicates that astrocytic thiol supply is able to modulate the neuronal stress response following proteasome inhibition and thereby rescuing neurons.

Finally, we re-visited the protective role of astrocytes on neurodegeneration triggered by the parkinsonian toxicant MPP^+^. The presence of astrocytes largely (100-fold) increased the non-toxic concentration range of MPP^+^ (Fig. 6d, e), and also conditioned medium supported neuroprotection (Fig. S17). Moreover, the MPP^+^-induced ATF-4 stress response, a hallmark of mitochondrial stress in neurons [15], was largely attenuated by astrocyte co-culture (Fig. 6f, Fig. S18). As for MG-132, direct addition of GSH to the mono-cultures prevented intracellular aggregate formation triggered by MPP^+^ or rotenone (Fig. S19). These data indicate a broad role of astrocytic thiol supply in the modulation of neuronal stress responses.

## Discussion

It has been known that any treatment that increases astrocytic NRF-2, leads to their release of thiols, which support neurons [21, 40–45]. However, it remained unclear how this works on the level of neurons. The data presented here link the numerous observations on astrocytic neuroprotection (and the regulation within astrocytes) to the hitherto little explored alterations in neuronal stress response signalling/cell death. Based on the established knowledge that neurons are in need of an extracellular thiol source [34, 36], and that astrocytes are the main suppliers of glutathione/glutathione precursors in the brain, we show here that this metabolic cooperation has a major impact on neurodegeneration. Using direct GSH supplementation, we found that changes in the regulation of key TF were linked to neuronal protection from insults as diverse as a block of the mitochondrial respiration chain and proteasome inhibition. Thiol supply did not dampen all stress responses elicited by proteasome inhibitors, but it rather led to a re-adjustment of cellular homoeostasis: while e.g. ATF-4 was strongly downregulated, the NRF-1 response was found to be increased. The overall shift of the response pattern appeared to be responsible for the change in neuronal fate (Fig. 5i). The findings of this study implicate astrocytes, and in particular the regulation of their thiol supply to neurons, as potential therapeutic drug targets.

Neurodegeneration in PD is closely linked to mitochondrial dysfunction [2, 6], and accordingly, many PD models rely on experimental inhibition of mitochondria [7]. A frequent observation in such models and human patients is that protein degradation is impaired [2, 6]. We found here that this link also pertains to the frequently used LUHMES neuronal cultures [15, 46–49]. Indeed, mitochondrial inhibition by two different compounds leads to an attenuation of proteasome activity, and to a subsequent accumulation of aggregated proteins. These mild, but significant effects on the UPS are well in line with those observed in animal studies [50]. Also, in agreement with many in vivo observations [8, 9], we found that direct inhibitors of the proteasome had more pronounced proteotoxic effects. Astrocytes attenuated neuronal cell death triggered by all toxicants used here, and we found the survival of cells with a specifically inhibited UPS particularly intriguing; as such, protection is not easily explained by the activation of known resilience pathways, such as a shifted energy supply [51] or the scavenging of ROS released from inhibited mitochondria [7]. As the cellular damage in the proteasome inhibition model is more clearly defined than in the mitochondrial dysfunction models, we mainly focused on the resilience mechanisms relevant for this form of damage, even though the neuroprotective principles may be applicable also to other toxicants, such as mitochondrial inhibitors.

Others have observed that addition of cysteine or an increase of GSH can attenuate the ATF-4 stress response and increase survival following proteasomal inhibition [16, 52]. From these studies, it has been concluded that proteasome activity is important to maintain intracellular cysteine levels in order to support protein biosynthesis [16]. Our data favour a different interpretation of the role of ATF-4 in human neurons: this TF appears to be a master controller of neuronal thiol supply by upregulating the cystine transporter and by enabling the transsulfuration pathway. The latter provides neurons with an alternative route of cysteine generation from methionine, and thus augments their glutathione levels and ROS resilience [53–55]. We have previously linked ATF-4 activation following mitochondrial respiratory chain inhibition to increased usage of the transsulfuration pathway in LUHMES, and we found that downregulation of ATF-4 reduced the GSH levels of stressed neurons dramatically [15]. This suggests that one consequence of ATF-4 upregulation, i.e. an augmented cellular GSH content, may be beneficial rather than detrimental. In accordance with this, we found ATF-4*−/−* cells to be rather more sensitive to MG-132 than the corresponding wild-type cells. We suggest that the drastic downregulation of neuronal ATF-4 by cysteine, GSH or astrocytes reflects a decreased need of neurons to activate the transsulfuration pathway in order to secure their GSH levels. However, the ATF-4 pathway has several branches that need to be considered. Besides its role in resilience under nutrient stress, it can also activate a cell death programme via induction of DDIT-4. We provided evidence that this branch plays a role in our model system. In the presence of thiols, added to the medium, or provided by astrocytes, this pro-death branch was blocked, while the protective branch was not necessary for the cells (transsulfuration is not required if cysteine supply is sufficient). We conclude that blunting of the ATF-4 response in the presence of additional GSH prevented an immediate execution of a death programme, but did not explain the coping of the cell with the primary problem of accumulated proteins.

Thus, the observed NRF-1 upregulation may be more critical. This TF has recently been identified as the main regulator of proteasome abundance [12, 28, 29]. In line with this, we observed the recovery of proteasome activity and reduced aggregate formation in cells supplemented with GSH. Alterations in NRF-1 levels by overexpression or knockdown increased or decreased cellular resilience against proteasome inhibition, respectively. Thus, extra supply of thiols to the neurons seemed to have two major effects: first, cells were prevented from immediately undergoing programmed cell death (decreased ATF-4/DDIT4/caspase axis); second, this first effect ‘bought sufficient time’ for NRF-1 to become active, and allow recovery of proteasome activity and clearance of aggregated proteins.

By linking astrocytic thiol supply to an increased NRF-1 response and survival of neurons, we identified astrocytes as the potential therapeutic target. One candidate pathway that may be targeted by drugs is the NRF-2 stress response. In brain tissue, astrocytes are the major cell type to activate this pathway [21, 56], and this response may be triggered by pharmacological or non-pharmacological pre-conditioning strategies [57, 58]. Stimulation or overexpression of NRF-2 in astrocytes leads to an intracellular GSH increase, to GSH release into the medium and to augmented protection of co-cultured neurons [21, 40, 41, 59, 60]. Moreover, some evidence suggests that activation of NRF-2 in astrocytes affects transcription in neurons [59]. The co-culture system established here may be used in the future to further characterise such effects and to examine drugs harnessing the endogenous transcellular resilience pathways for neuroprotection.

## Materials and methods

### Chemicals

Dibutyryl-cAMP (cAMP), fibronectin, Hoechst bisbenzimide H-33342, resazurin sodium salt, tetracycline, L-cysteine and reduced glutathione (GSH) were purchased from Sigma (Steinheim, Germany). Recombinant human FGF-2 and recombinant human GDNF were purchased from R&D Systems (Minneapolis, USA). Tween-20 and sodium dodecyl sulphate (SDS) were purchased from Roth (Karlsruhe, Germany). All cell culture reagents were purchased from Gibco/Fisher Scientific (Hampton, New Hampshire, USA) unless otherwise specified. MG-132 was purchased from Selleckchem (Houston, USA).

### Cell culture

Handling of LUHMES human neuronal precursor cells was performed as previously described in detail [25]. Briefly, the conditionally immortalised cells, maintained in proliferation medium, consisting of advanced DMEM/F12, 2 mM L-glutamine, 1 x N2 supplement (Invitrogen) and 40 ng/ml FGF-2 in a 5% CO_2_/95% air atmosphere at 37 °C, were passaged every other day. For differentiation, 8 million cells were seeded in a Nunclon T175 tissue culture flask pre-coated with 50 µg/ml poly-L-ornithine (PLO) and 1 µg/ml fibronectin in proliferation medium. After 24 h, the medium was changed to differentiation medium (DM), consisting of advanced DMEM/F12 supplemented with 2 mM L-glutamine, 1 x N2, 2.25 µM tetracycline, 1 mM dibutyryl 3′,5′-cyclic adenosine monophosphate (cAMP) and 2 ng/ml recombinant human glial cell-derived neurotrophic factor (GDNF). After 48 h, cells were trypsinised and seeded at a density of 1.5 × 10^5^ cells/cm² into dishes pre-coated with 50 µg/ml poly-L-ornithine (PLO) and 1 µg/ml fibronectin in DM. The medium of maintenance was regularly checked for mycoplasma contamination and a working stock of LUHMES cells was validated by STR profiling to exclude cross-contamination.

Astrocytes generated from mouse embryonic stem cells (mAGES) were differentiated, as described previously [17, 27]. Briefly, neural stem cells (NSC) were cultivated in N2B27 medium supplemented with 20 ng/ml FGF2 and 20 ng/ml EGF. For differentiation to mAGES, FGF2 and EGF were replaced with 20 ng/ml BMP4 (R&D Systems, Minneapolis, USA) for at least 3 days. The human iPSC-derived astrocytes Astro.4U (Ncardia, Köln, Germany) were thawed according to the manufacturer’s instructions and cultivated in Astro.4U medium on Matrigel-coated plates. For astrocyteneuron co-cultures, pre-differentiated LUHMES (d2) were seeded either on top of mAGES (day 3) or onto Astro.4U (9 days after thawing).

Human embryonic kidney cells 293 (HEK-293) were cultured in DMEM (Gibco/Fisher Scientific, Hampton, New Hampshire, USA) supplemented with 10% foetal calf serum and 1% pen/strep. Cells were passaged every other day. For viability experiments, cells were seeded 24 h prior to the start of the experiment at a density of 7 × 10^4^ cells/cm².

### Astrocyte-conditioned medium

The medium of differentiated astrocytes was aspirated and cells were washed with PBS, and then LUHMES DM was added and the astrocytes were cultivated in this medium for 72 h. After the incubation, the medium was harvested, centrifuged for 5 min at 350 × *g*, to remove cell debris and added to differentiated LUHMES (d6) cells prior to the start of the experiment.

### General cell viability endpoints

#### Resazurin

Metabolic activity was detected by a resazurin assay. Briefly, resazurin solution was added to the cell culture medium to obtain a final concentration of 10 µg/ml. After incubation, for 30 min at 37 °C, the fluorescence signal was measured at an excitation wavelength of 530 nm, using a 590-nm long-pass filter to record the emission. Fluorescence values were normalised by setting the fluorescence values of untreated wells as 100%.

#### LDH release

LDH activity was detected separately in the supernatant and in the corresponding cell homogenate. The medium was transferred into a separate plate, and then the cells were lysed in PBS/0.1% Triton X-100 for 2 h. Twenty microlitres of sample was added to 180 μl of reaction buffer containing NADH (100 μM) and sodium pyruvate (600 μM) in potassium-phosphate buffer (pH 7.4). Absorption at 340 nm was measured at 37 °C in 1-min intervals over a period of 15 min. The slope of NADH consumption was calculated. The ratio of LDH_supernatant_/LDH_total_ was calculated using the slopes of supernatant and homogenate. LDH release was expressed in percent. Control data were subtracted from LDH values.

### Specific neuronal viability assay (neurite area)

#### Calcein-AM

Labelling of live cells was performed with 1 µM calcein-AM/1 µg/ml H-33342 for 30 min at 37 °C. Images were collected in two different fluorescent channels using an automated microscope (Array-Scan VTI HCS Reader (Thermo Fisher, PA, USA)). Using an imaging software (vHCS SCAN, Thermo Fisher, PA, USA), nuclei were identified in channel 1 (365 ± 50/461 ± 15 nm) as objects according to their size, area, shape and intensity. Calcein signal was detected in channel 2 (475 ± 40/525 ± 15 nm). An algorithm quantified all calcein-positive cells as viable and only H-33342-positive nuclei as 'not viable' cells. For evaluating the neurite areas, nuclei masks, determined in channel 1, were expanded and transferred to channel 2. All calcein-positive pixels outside of these masks (somatic area) were counted as neurite area.

### Determination of total glutathione

For glutathione determination, cells were washed with PBS and lysed in 400 μl of 1% sulphosalicylic acid (w/v). The lysates were collected, sonicated five times and centrifuged at 12,000 × *g*, 5 min, 4 °C to remove cell debris. The total glutathione content was determined by a DTNB (5,5′-dithiobis(2-nitrobenzoic acid)) reduction assay. In total, 20 μl of sample was mixed with 180 μl of assay mixture containing 300 μM DTNB, 1 U/ml glutathione reductase, 400 μM NADPH and 1 mM EDTA in 100 mM sodium phosphate buffer, pH 7.5 (all purchased from Sigma, Steinheim, Germany). DTNB reduction was measured photometrically at 405 nm in 5-min intervals over 30 min. GSH standard curves were performed by serial dilutions ranging from 1000 to 7.8 nM, respectively.

### Western blot analysis

Cells were lysed in 1x Laemmli buffer and boiled for 5 min at 95 °C. For removal of long DNA strands, lysates were centrifuged for 1 min, 10,000 × *g* using NucleoSpin Filters (Macherey-Nagel, Düren, Germany). Thirty-five micrograms of total protein were loaded onto 6–15% SDS gels according to the size of the protein of interest. Proteins were transferred onto nitrocellulose membranes (Amersham, Buckinghamshire, UK) using the Invitrogen iBlot 2 system. Membranes were blocked with 5% BSA (w/v) in TBS-Tween (0.1% (v/v)) for 1 h. Primary antibodies were incubated at 4 °C overnight. Following the washing steps with TBS-Tween (0.1%), horseradish peroxidase-conjugated secondary antibodies were incubated for 1 h at RT. For visualisation, ECL western blotting substrate (Pierce/Thermo Fisher Scientific, Rockford, IL, USA) was used. Antibodies used for western blot analysis are specified in Fig. S20. The relative band intensities were quantified using dedicated image quantification software. Band intensities were normalised to the corresponding GAPDH controls. Data are expressed as fold-induction relative to untreated.

### Immunocytochemistry

Cells were grown on 13mm glass coverslips (Menzel, Braunschweig, Germany) in 24-well plastic cell culture plates (Nunclon^TM^) and fixed with 4% paraformaldehyde. After incubation with the primary antibody overnight and with the appropriate secondary antibody for 1 h, Hoechst-33342 (1 µg/ml) was added for 10 min prior to the final washing step. Coverslips were mounted on glass slides with Fluorsave reagent (Calbiochem/Millipore/Darmstadt/Germany). For visualisation, an Olympus IX81 inverted epifluorescence microscope (Hamburg, Germany) was used. For image processing, Image J open-source software was used.

### Detection of apoptosis

Cells were stained with antibodies against cleaved caspase 3 and β-III-tubulin. Images were collected in three different fluorescent channels using an automated microscope (Array-Scan VTI HCS Reader (Thermo Fisher, PA, USA)). Using an imaging software (vHCS SCAN, Thermo Fisher, PA, USA), nuclei were identified in channel 1 (365 ± 50/461 ± 15 nm) as objects according to their size, area, shape and intensity. Cleaved caspase 3 signal was detected in channel 2 (475 ± 40/525 ± 15 nm). An algorithm quantified all cleaved caspase 3-positive cells as apoptotic and only H-33342-positive nuclei as cells.

### Detection of protein aggregates

For detection of protein aggregates, cells were treated with toxicants for the time period as indicated, fixed with 4% paraformaldehyde and stained with PROTEOSTAT Aggresome detection kit (ENZO, Lausen, Switzerland) following the manufacturer’s instructions. Images were collected in two different fluorescent channels using an automated microscope (Array-Scan VTI HCS Reader (Thermo Fisher, PA, USA)). Using an imaging software (vHCS SCAN, Thermo Fisher, PA, USA), nuclei were identified in channel 1 as objects according to their size, area, shape and intensity. PROTEOSTAT dye signal was detected in channel 2. For evaluating the intensity of PROTEOSTAT dye, the intensity in channel 2 was quantified.

### Detection of proteasome activity

For detection of proteasome activity, cleaveage of MeOSuc-Gly-Leu-Phe-AMC (Bachem, Bubendorf, Switzerland) was measured fluorometrically. Cells were treated with test compounds for the time periods, as indicated, as a positive control MG-132 [10 µM] was added 4 h prior to measurement of proteasome activity. The medium was replaced with HBSS containing MeOSuc-Gly-Leu-Phe-AMC [25 µM] and fluorescence was measured (ex: 360 nm, em: 465 nm) directly and after 1 h of incubation.

### Quantitative PCR (qPCR)

For reverse transcription quantitative PCR (RT-qPCR) analysis, RNA was extracted with the PureLink RNA mini Kit (Invitrogen, Darmstadt, Germany) according to the manufacturer’s instructions. For transcript analyses of LUHMES, primers (Eurofins MWG Operon, Ebersberg, Germany) were designed as described in ref. [15] and can be found in Fig. S21. All RT-qPCRs were based on the SsoFast EvaGreen detection system and were run in a CFX96 Cycler (Biorad, München, Germany) and analysed with Biorad iCycler software. The threshold cycles (Ct) were determined for each gene and gene expression levels were calculated as relative expression compared to GAPDH (2^−(ΔCt)^) or as fold change relative to control (2^−(ΔΔCt)^). ΔCt and ΔΔCt were calculated according to the following formulas:

ΔCt = Ct(condition_gene Y) – Ct(condition_GAPDH).

ΔΔCt = ΔCt(condition_gene Y) – ΔCt(untreated control_gene Y).

gene Y = gene of interest; GAPDH was used as a housekeeping gene for normalisation.

### Amino acid analysis

Twelve million cells were washed once with PBS and quenched with 50% v/v methanol/H_2_O. After shaking for 30 min at 1400 rpm at 4 °C in an Eppendorf Thermomix (Hamburg, Germany), the solution was centrifuged for 15 min at 21,000 × *g* at 4 °C to separate the supernatant from the protein precipitations. Samples were dried in a speedvac concentrator and reconstituted in 135 µl of sample dilution buffer (pH 2.2, 0.12 M) (Sykam, Fürstenfeldbruck, Germany). The amino acids were then quantified using a Sykam S433 Amino acid analyser (Sykam, Fürstenfeldbruck, Germany). Shortly, amino acids and ammonia were separated by HPLC and subsequent post-column derivatisation with ninhydrin. Samples were injected in a volume of 100 µl. Chromatography was performed using a lithium-based anion exchange column loaded with a spherical polystyrene resin (7 µm diameter, 10% cross-links, cat# 5125022). Elution was performed using buffers with increasing pH and ion strength (pH 2.9→pH 12; buffer concentration 0.12-0.45 M), supported by a temperature gradient. Absorbance of the reaction products was quantified at 440 nm (an intermediate product quantifies cysteine and proline) or 570 nm (quantifies all other amino acids). Amino acid concentrations were determined relative to a reference standard using the area under the peak method in the ChromStar 7 software (SCPA, Weyhe-Leehste, Germany) [51].

### Statistics and data mining

Data are presented as means of at least three independent experiments, unless otherwise indicated. Error bars are used to indicate data variation; in addition, individual data points are displayed where this makes the data structure more transparent. Statistical differences were tested using GraphPad Prism 5.0 (GraphPad Software, La Jolla, USA); the type of error bar, the test used and the post hoc comparison approaches employed are indicated in the figure legends. Apparent molecular weights of bands in western blots are indicated based on the position of molecular weight markers run on the same gels.

## Electronic supplementary material

Supplement Figures Astrocytic neuroprotection
