# Supplementary material for: Prevention of neuronal apoptosis by astrocytes through thiol-mediated stress response modulation and accelerated recovery from proteotoxic stress
Source: Cell Death Differ. 2018 Nov 2;25(12):2101–17. doi: 10.1038/s41418-018-0229-x (PMC6261954; doi:10.1038/s41418-018-0229-x)
Supplement: Supplementary file 1 — Supplement Figures Astrocytic neuroprotection [file 41418_2018_229_MOESM1_ESM.pdf]

Supplementary Information

Prevention of neuronal apoptosis by astrocytes through thiol-mediated stress response modulation and accelerated recovery from proteotoxic stress

Simon Gutbier[1,5], Anna-Sophie Spreng[1,6], Johannes Delp[1,5,7], Stefan Schildknecht[1], Christiaan Karreman[1], Ilinca Suciu[1,6], Thomas Brunner[2], Marcus Groettrup[3] and Marcel Leist[1,4]

Overview:

Data figures (Fig. S1-19) are shown on pages 2-15; Lists of antibodies (Fig. S20) and primers (Fig. S21) are indicated on pages 16+17. References from the figure legends are on page 18.

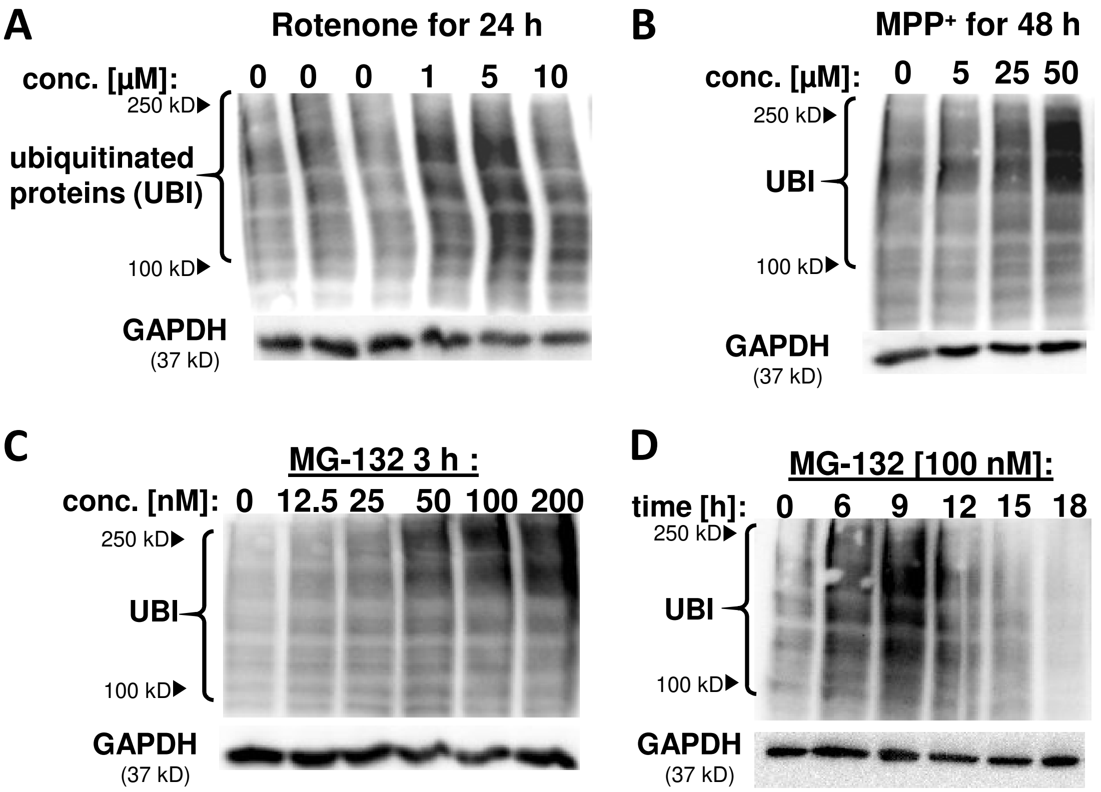

Fig. S1: Accumulation of ubiquitinated proteins (UBI) by different stressors

A/B/C: To test for proteasomal dysfunction, cells were treated with the indicated concentrations of rotenone for 24 h, or MPP^+ for 48 h or MG-132 for 3 h. The caspase inhibitor Q-VD-Oph [5 µM] was used as media supplement to prevent cells from dying. Then, cells were lysed and analysed by Western blot with anti-ubiquitin and anti-GAPDH (loading control) antibodies .

D: To confirm proteasomal dysfunction, cells were treated with 100 nM MG-132 for the indicated time periods. After incubation cells were lysed and analysed by Western blot with anti-ubiquitin and anti-GAPDH antibodies.

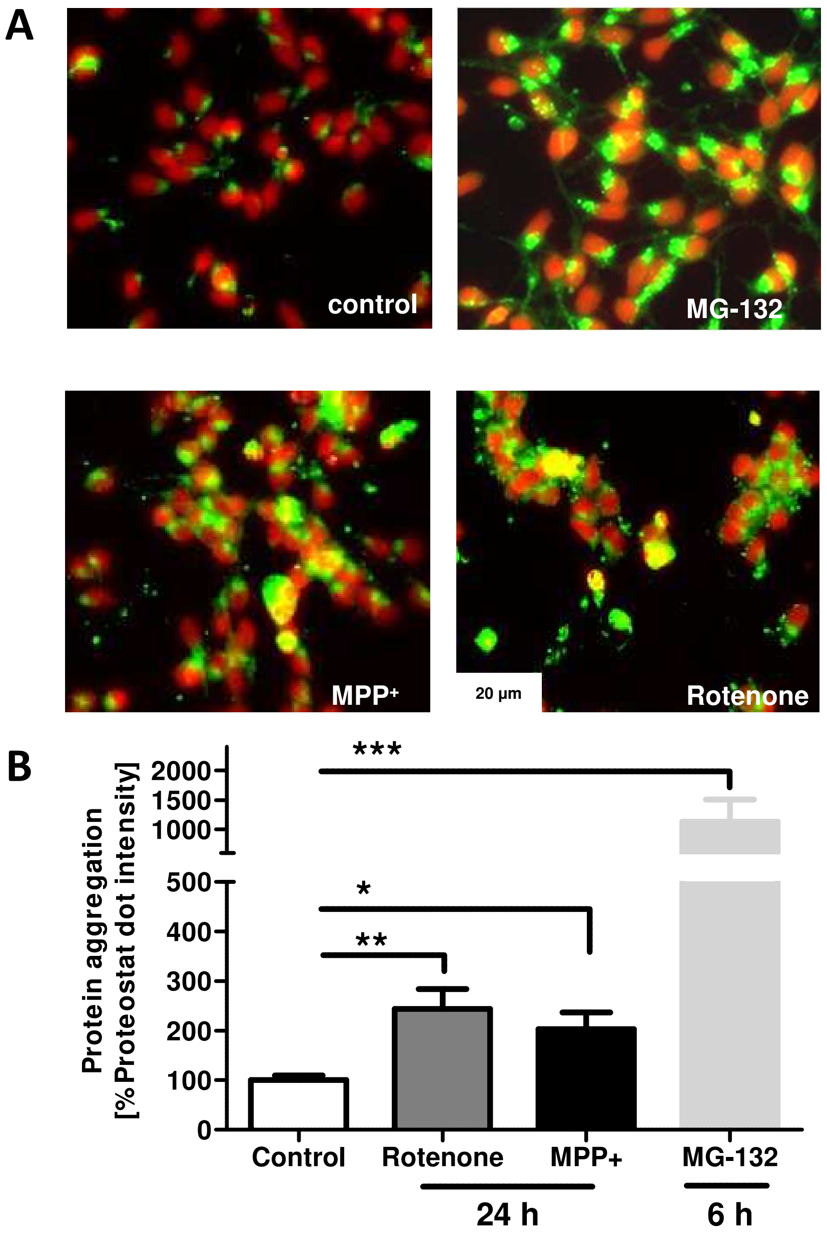

Fig. S2: Accumulation of protein aggregates in neurons

A: To test for proteasomal dysfunction, cells were treated with either rotenone [1 µM] or MPP^+ [5 µM] for 24 h or with MG-132 [100 nM] for 6 h. Then, cells were fixed with paraformaldehyde (PFA), stained with Proteostat detection reagent (Enzo, Lausen, Switzerland) based on a dye binding to β-sheet protein aggregates, and analysed by automated microscopy. Aggregates are displayed in green, DNA (nuclei) are shown in red.
B: Quantification of fluorescence intensity of the Proteostat reagent (measuring protein aggregates) in the samples described in A. Data are means ± SEM (N=3); *: $p < 0.05$, **: $p < 0.01$, ***: $p < 0.001$ (ANOVA followed by Dunnett's post-hoc test).

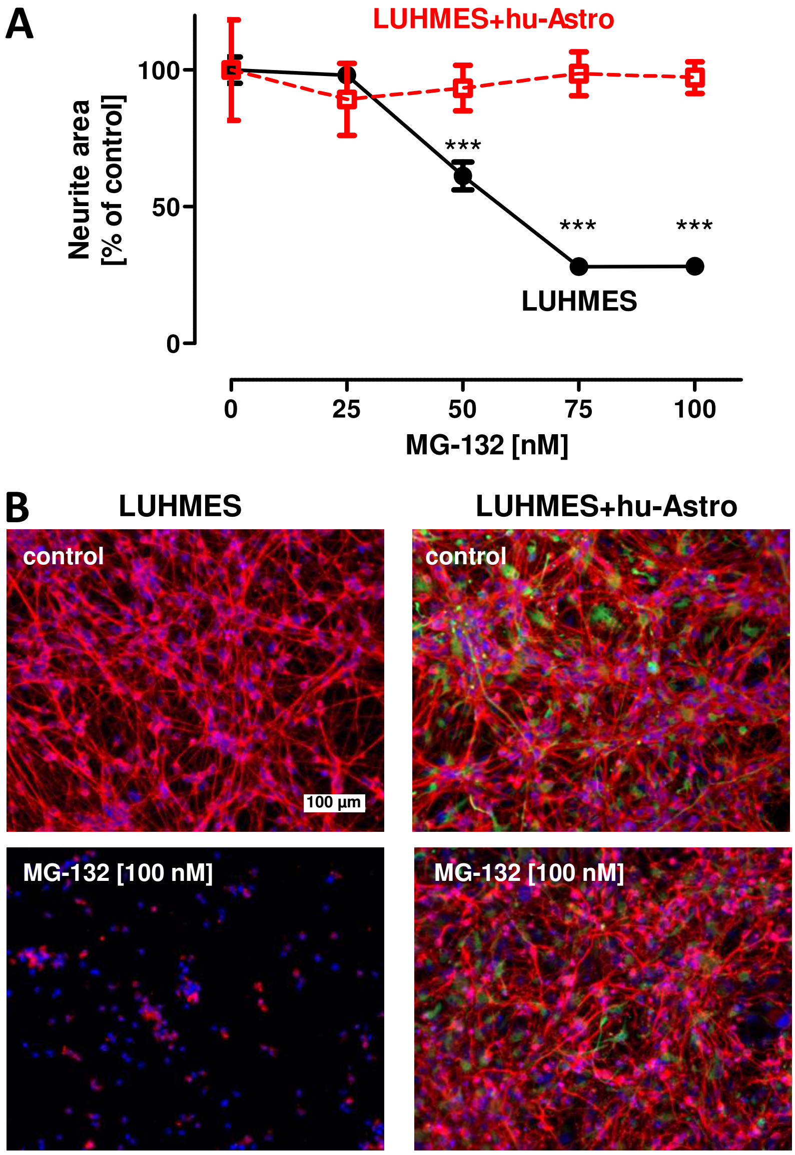

Fig. S3: Neuronal protection by human stem cell derived astrocytes (hu-Astro)
Human astrocytes (hu-Astro) were differentiated from induced pluripotent stem cells following established protocols (Chandrasekaran 2016). The cells were obtained as Astro.4U from Ncardia (Cologne, Germany) and plated according to manufacturer's instructions. More than 85% of the cells were GFAP positive (immunostaining). LUHMES were plated on top of the cells as described earlier (Efremova 2016).
A: Differential toxicity of MG-132 in co- and mono-culture was assessed by immunocytochemistry staining against β-III tubulin, GFAP and H-33342 after cultures were exposed for 24 h to MG-132 at the indicated concentrations. Toxicity of MG-132 on LUHMES and astrocyte-LUHMES co-culture was assessed by measuring the neurite integrity after cells were exposed for 24 h to MG-132 at the indicated concentrations. Data are means ± SD of three independent experiments, ***: $p<0.001$. **B**: Exemplary images of the different experimental conditions are displayed for the 24 h time point.

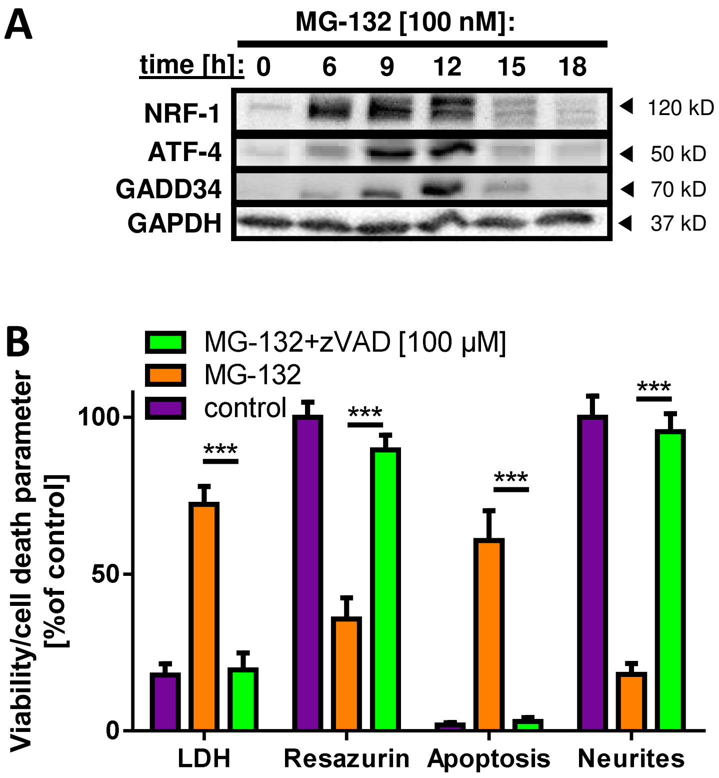

Fig. S4: Stress response and cell death signals triggered by MG-132 in neurons

A: To analyse the stress response following proteasome inhibition, cells were treated with 100 nM MG-132 for the indicated time periods. After incubation, cells were lysed and analysed by Western blot with anti-NRF-1, anti-ATF-4, anti-GADD34 and anti-GAPDH antibodies. The blots shown are representative for three experiments with similar results.

B: LUHMES (d6) cells were exposed to MG-132 [100 nM] for 20 h in the presence or absence of z-Vad-fmk [100 µM]. The different cell death/viability endpoints were measured. LDH-release is indicated as the percentage of total enzymatic activity in the well that is found in the media supernatants. Resazurin data reflects viable cells (100% corresponds to all cells are viable). Apoptosis refers to counting of nuclei with apoptotic morphology relative to all nuclei. Neurites refers to the integrity of the neuronal network as determined by high content imaging. It is a viability measure (100% = healthy cells) like resazurin. Data were analysed for statistical differences between treatments by two-way ANOVA, followed by a Dunnett's post-hoc test, ***: $p < 0.001$.

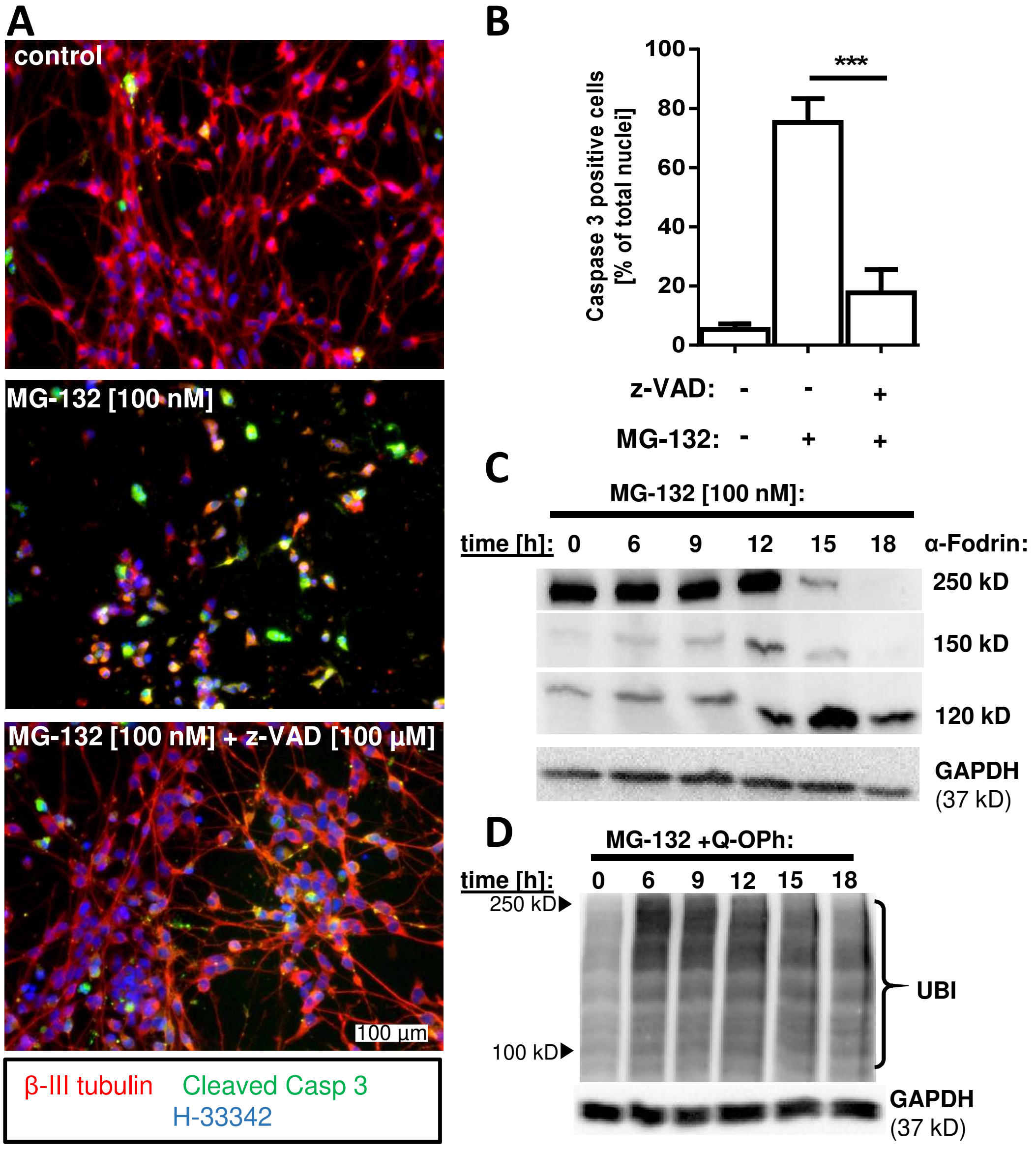

Fig. S5: Induction of caspases by treatment of neurons with MG-132

A: Differentiated LUHMES (d6) cells were exposed to either solvent, 100 nM MG-132 or 100 nM MG-132 plus 100 µM caspase inhibitor z-VAD for 18 h. After incubation, cells were fixed and immunostaining for β-III tubulin, cleaved caspase 3 (Casp3) and the DNA dye H-33342 was performed. Representative pictures for each condition are shown. **B:** Cells immunostained as in A were analysed for cleaved caspase 3 positive cells by automated microscopy using a scoring algorithm that counts Hoechst and cleaved caspase 3 double positive nuclei. Data are means ± SD from three independent experiments. Data were analyzed for significant differences between treatments by one-way ANOVA and a Bonferroni post-hoc test, ***: $p<0.001$. **C:** To confirm caspase activity, cells were treated with 100 nM MG-132 for the indicated time periods. After incubation, cells were lysed and analysed by Western blot with anti-Fodrin and anti-GAPDH antibodies. **D:** To test for proteasomal dysfunction and degradation of ubiquitinated proteins by caspases, cells were treated with MG-132 [100 nM] for the indicated time periods. The caspase inhibitor Q-VD-Oph [5 µM] was used as media supplement to prevent cells from dying and to test the effect of caspase inhibitor on ubiquitination. After incubation, cells were lysed and analysed by Western blot with anti-Ubiquitin and anti-GAPDH antibodies.

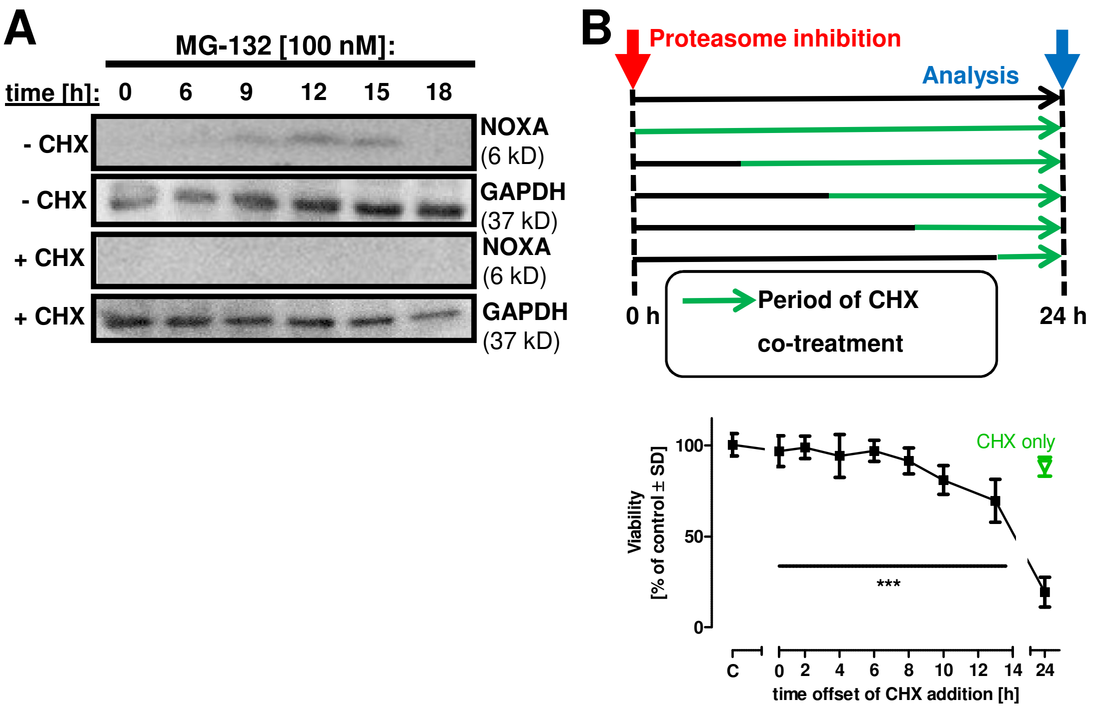

Fig. S6: Prevention of MG-132 induced neuronal apoptosis by the inhibition of protein synthesis

A: LUHMES cells (d6) were treated with MG-132 [100 nM] in presence or absence of cycloheximide (CHX) [10 µM] for the indicated time periods. After incubation, neurons were lysed and analysed by Western blot with anti-NOXA and anti-GAPDH antibodies. **B:** LUHMES cells (d6) were treated with MG-132 [100 nM], and CHX [10 µM] was added at various indicated time points after the start of MG-132 exposure. Viability was assessed using calcein-AM/H-33342 staining at 24 h after start of the MG-132 exposure. Double positive cells were counted by automated microscopy and normalized for all H-33342 positive cells. Differences were tested for significance (N= 3 experiments) by one-way ANOVA followed by Dunnett's post-hoc test, ***: $p<0.001$ for comparison of samples with CHX added vs MG-132 treatment without CHX (= 24 h data point). The green triangle indicates the viability of cells treated with only cycloheximide for 24 h.

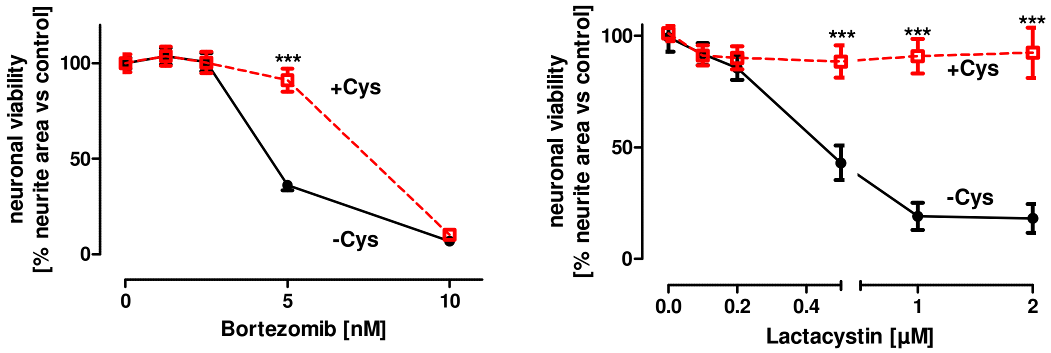

Fig. S7: Protective effect of cysteine against different proteasome inhibitors

Differentiated (d6) LUHMES cells were exposed to indicated concentrations of bortezomib (A) or lactacystin (B) in the presence or absence of L-cysteine [1 mM]. Neuronal viability was assessed after 24 h of exposure by measuring the calcein positive neurite area. Data are means ± SD of three independent experiments. ***: p<0.001.

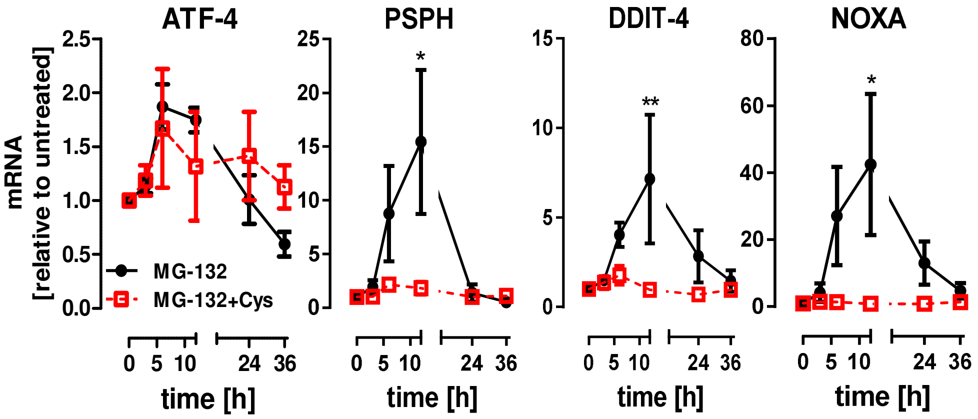

Fig. S8: Induction of ATF-4 target genes by MG-132

Differentiated (d6) LUHMES cells were exposed to MG-132 [100 nM] for the indicated time periods in the presence or absence of L-cysteine [1 mM]. Changes in mRNA levels were monitored by qPCR for activating transcription factor 4 (ATF-4) and its target genes phosphoserine phosphatase (PSPH), DNA damage inducible transcript 4 (DDIT-4) and phorbol-12-myristate-13-acetate-induced protein 1 (NOXA). Data are means ± SEM of three independent experiments. Detected differences were tested for significance by two-way ANOVA comparing the time points of the different treatments, followed by a Bonferroni post-hoc test to correct for multiple comparisons, *: p<0.05 **: p<0.01; ***: p<0.001.

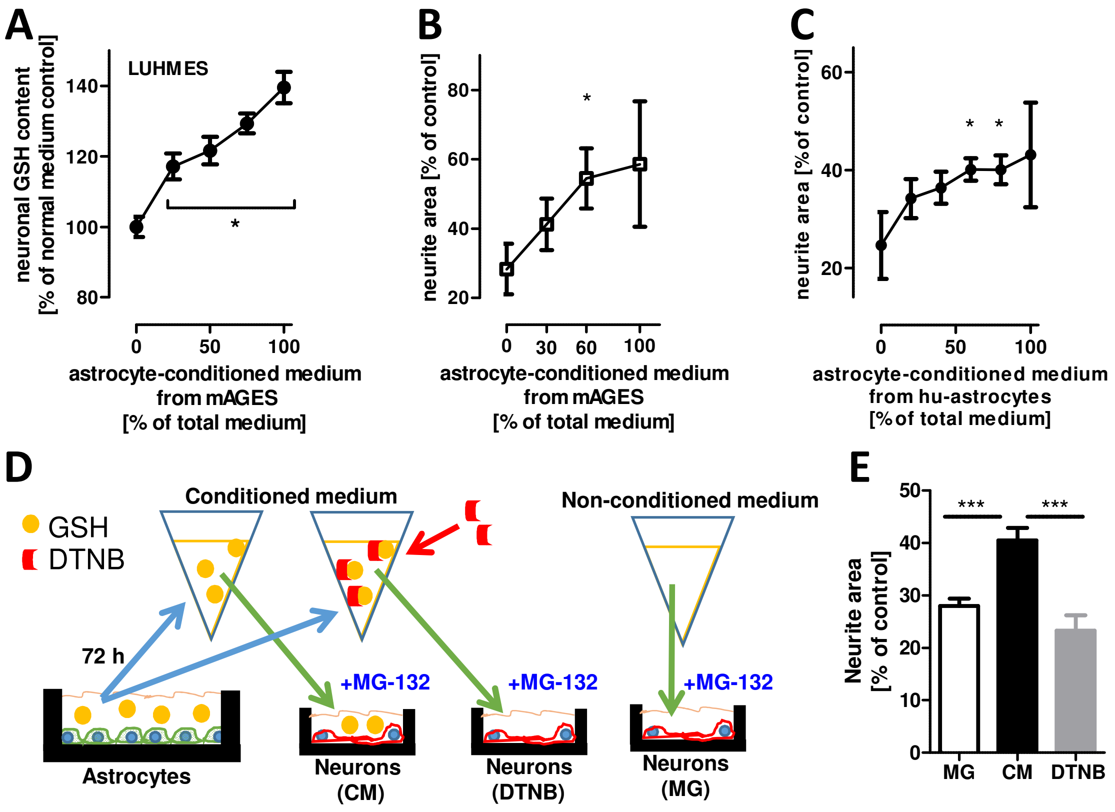

Fig. S9: Elevation of neuronal GSH and protection against MG-132 by astrocyte-conditioned medium

A: Differentiated (d6) LUHMES cells were exposed for 4 h to astrocyte-conditioned medium, diluted with fresh LUHMES culture medium. Neuronal intracellular total glutathione (GSH+GSSG) was measured after incubation. Data are means ± SD of three independent experiments. **B:** Differentiated (d6) LUHMES cells were exposed to astrocyte-conditioned medium from mAGES, diluted with fresh LUHMES culture medium in the presence or absence of MG-132 [100 nM]. Viability was assessed 24 h after start of MG-132 exposure. For measurement of viability, cells were stained with the vital dye calcein-AM and the DNA stain H-33342. Neurite area of the cells was assessed by automated microscopy. Data are means ± SD of three independent experiments. **C:** Differentiated (d6) LUHMES cells were exposed to astrocyte-conditioned medium from hu-Astro (see Fig. S3) in the presence or absence of MG-132 [100 nM]. Viability was assessed as in B. Data are means ± SD of three independent experiments. For A-C, the significance of individual data points was tested by ANOVA followed by Dunnett's post-hoc test (*: $p < 0.05$). In addition, all curves showed a significant overall rising trend ($p < 0.05$ for slope non-equal to zero). **D:** Experimental setup of thiol scavanging. Astrocyte conditioned medium from mAGES was harvested and treated either with 10 µM DTNB (5,5-dithio-bis-2-nitrobenzoic acid/ Ellman's reagent) or solvent control. Conditioned medium was transferred onto LUHMES cells. They were treated with MG-132 [100 nM] for 18 h. In parallel, LUHMES were incubated with un-conditioned medium and treated with MG-132 [100 nM] for 18 h. **E:** For the conditions described in D, viability of LUHMES was measured as in B. In parallel it was controlled that DTNB alone had no effect on neurite area (data not shown). Data are means ± SEM of three independent experiments, ***: $p<0.001$ (ANOVA followed by Dunnett's post-hoc test).

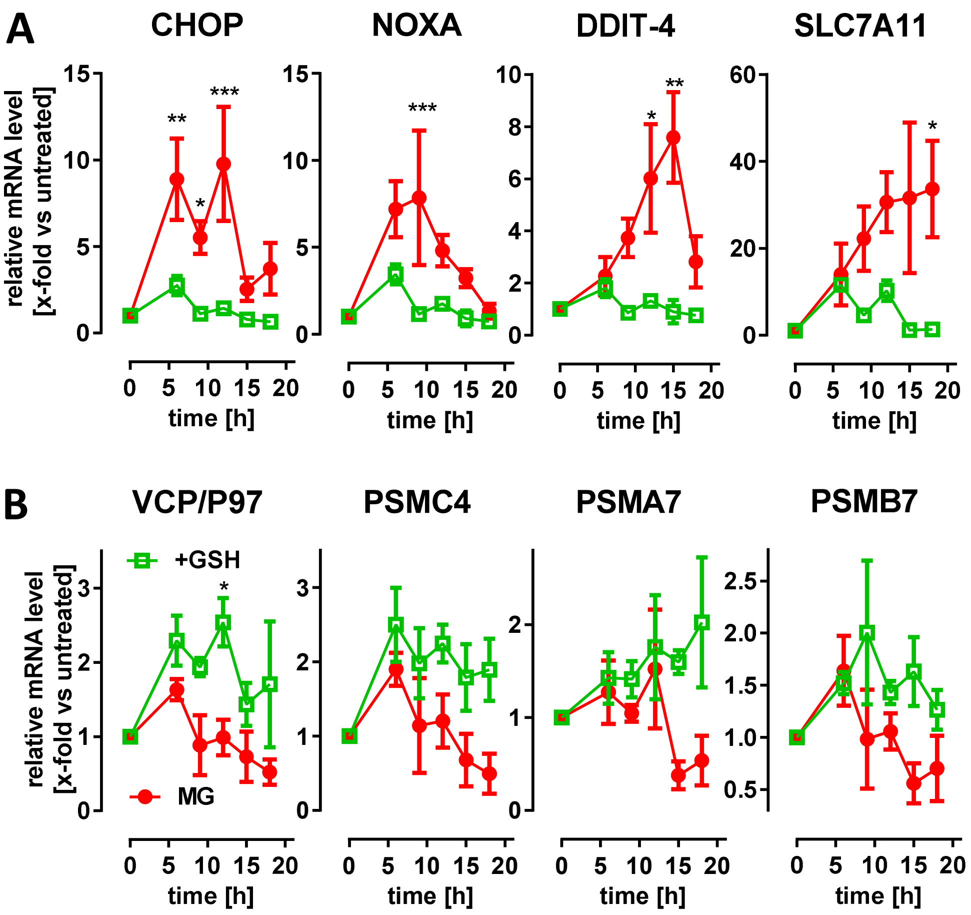

Fig. S10: Differences in the induction of ATF-4 and NRF-1 target genes by MG-132 in presence or absence of GSH

A: Differentiated d6 LUHMES cells were exposed to MG-132 [100 nM] for the indicated time periods in the presence or absence of GSH [1 mM]. Changes in mRNA levels were monitored by qPCR for the target genes of activating transcription factor 4 (ATF-4): cystine transporter (SLC7A11), DNA damage inducible transcript 4 (DDIT-4), Phorbol-12-Myristate-13-Acetate-Induced Protein 1 (NOXA) and DNA damage inducible transcript 3 (CHOP). **B:** Differentiated day 6 LUHMES cells were exposed to MG-132 [100 nM] for the indicated time periods in the presence or absence of GSH [1 mM]. Changes in mRNA levels were monitored by qPCR for the target genes of Nuclear Factor (Erythroid-Derived 2)-Like1(NRF1): valosin containing protein (VCP/p97), proteasome 26S subunit ATPase 4 (PSMC4), proteasome subunit alpha 7 (PSMA7) and proteasome subunit beta 7 (PSMB7). Data are means ± SEM of three independent experiments. Differences were tested for significance by two-way ANOVA comparing the time points of the different treatments, followed by a Bonferroni post-hoc test to correct for multiple comparisons, *: $p<0.05$ **: $p<0.01$; ***: $p<0.001$.

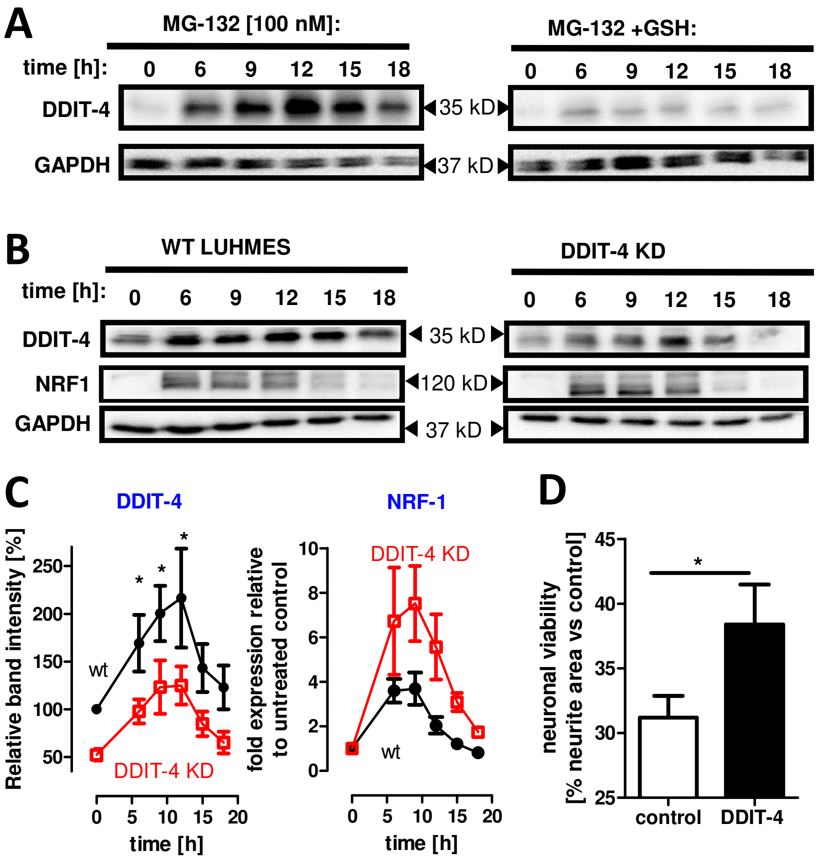

Fig. S11: Differences in the induction of ATF-4 target DDIT-4 by MG-132 in presence or absence of GSH

A:To address differences in the neuronal stress response following proteasome inhibition in the absence or presence of GSH [1 mM], cells were treated with MG-132 [100 nM] for the indicated time periods. After incubation, cells were lysed and analysed by Western blot using anti-DDIT4 and anti-GAPDH antibodies. **B:** LUHMES wilde type cells (wt) and DDIT-4 knock down (KD) cells were incubated with MG-132 [100 nM] for indicated time periods. After incubation, cells were lysed and analysed by Western blot using anti-DDIT-4, anti-NRF-1 and anti-GAPDH antibodies. **C:** Densitometric quantification of the DDIT-4 and the NRF-1 stress response following MG-132 exposure. Data are means ± SEM of three independent experiments. Differences were tested for significance by two-way ANOVA comparing the time points of the different treatments, followed by a Bonferroni post-hoc test to correct for multiple comparisons, *: $p<0.05$ **: $p<0.01$; ***: $p<0.001$. **D:** Differentiated (d6) LUHMES wild type and DDIT-4 knock down cells were exposed MG-132 [100 nM] for 18 h. Neurite viability was assessed by measuring the calcein positive neurite area. Data are means ± SD of three independent experiments, *: $p<0.05$ (*t*-test).

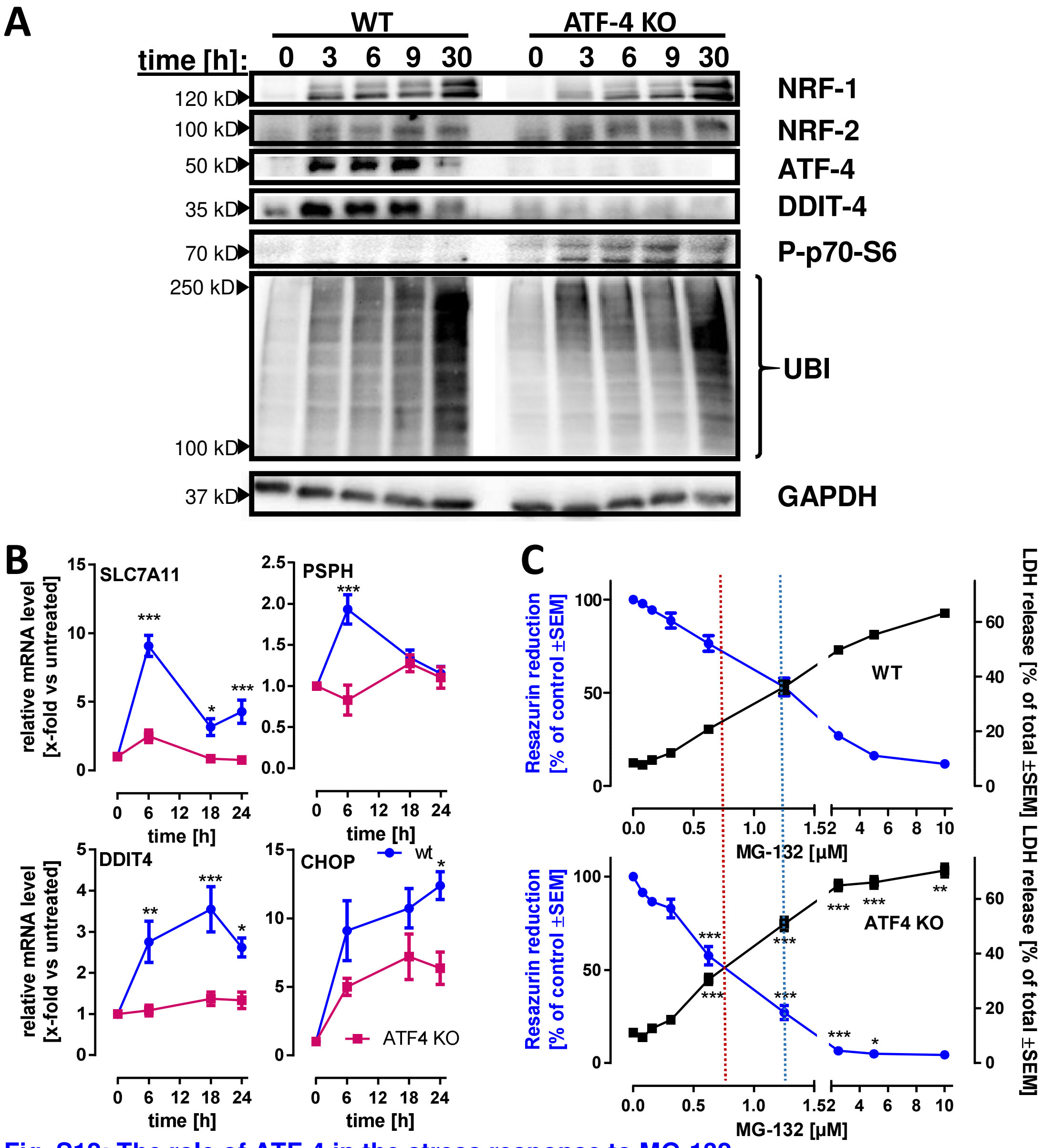

Fig. S12: The role of ATF-4 in the stress response to MG-132

To address the role of ATF-4 in the stress response following MG-132, we generated an ATF-4 knockout clone of HEK-293 cells using the CrispR/Cas9 system. For generation of the knockout, cells were transfected with the CREB-2 CRISPR/CAS9 KO Plasmid mix from Santa Cruz (sc-400155) and the CREB-2 HDR Plasmid (sc-400155-HDR). 24 h after transfection, cells were selected with puromycin [0.5 µg/ml] for 48 h. After selection, cells were plated by limiting dilution to obtain single cell clones. Several clones were assessed by Western blot for the absence of ATF-4 after MG-132 treatment. Three clones displaying no ATF-4 signal were further characterized. ATF-4 k.o. cells compared to wild type showed a decrease in growth rate, but looked morphologically normal. **A:** ATF-4 k.o. and wildtype cells were incubated with MG-132 [0.5 µM] for indicated time periods. After incubation, cells were lysed and analysed by Western blot using anti-ATF-4, anti-NRF-1, anti NRF-2, anti-DDIT-4, anti- phospho-p70S6, anti-ubiqitin and anti-GAPH antibodies **B:** ATF-4 k.o. and wildtype cells were incubated with MG-132 [0.5 µM] for indicated time periods. The mRNA levels for the ATF-4 target genes SLC7A11, PSPH, DDIT4 and CHOP were assessed using RT-qPCR. Differences were tested for significance by two-way ANOVA comparing the time points of the different treatments, followed by a Bonferroni post-hoc test, *: p<0.05 **: p<0.01; ***: p<0.001. **C:** ATF-4 k.o. and wildtype cells were incubated for 48 h with MG-132 at the indicated concentrations. Viability was assessed by resazurin and LDH release measurement. Data are means ± SD of three independent experiments. Differences between ATF-4 k.o. and wildtype cells were tested for significance by two-way ANOVA comparing the different concentrations, followed by a Bonferroni post-hoc test, *: p<0.05 **: p<0.01; ***: p<0.001.

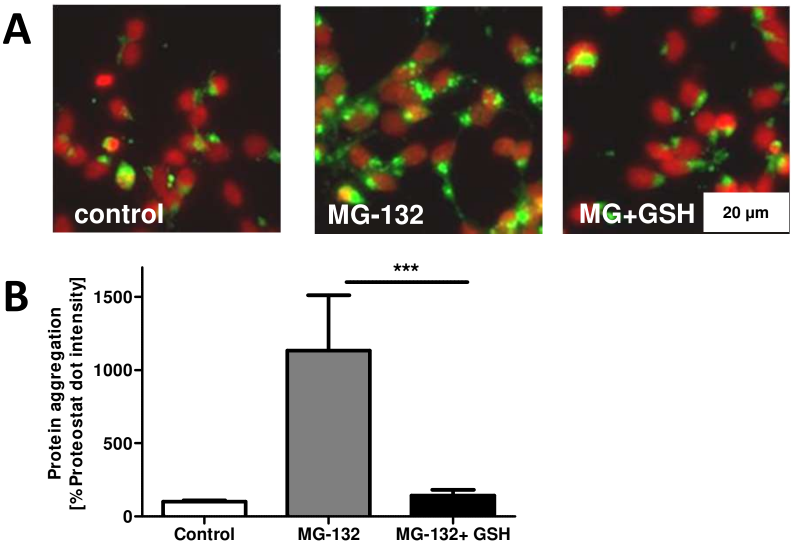

Fig. S13: Influence of GSH on the accumulation of protein aggregates in neurons

A: To test for proteasomal dysfunction, cells were treated with MG-132 [100 nM] for 6 h in the presence or absence of GSH [1 mM]. Then, cells were fixed with paraformaldehyde (PFA), stained with Proteostat detection reagent (Enzo, Lausen, Switzerland) based on a dye binding to β-sheet protein aggregates, and analysed by automated microscopy. Aggregates are displayed in green, DNA (nuclei) are shown in red.

B: Quantification of fluorescence intensity of the Proteostat reagent (measuring protein aggregates) in the samples described in A. Data are means + SD of three independent experiments, ***: $p<0.001$.

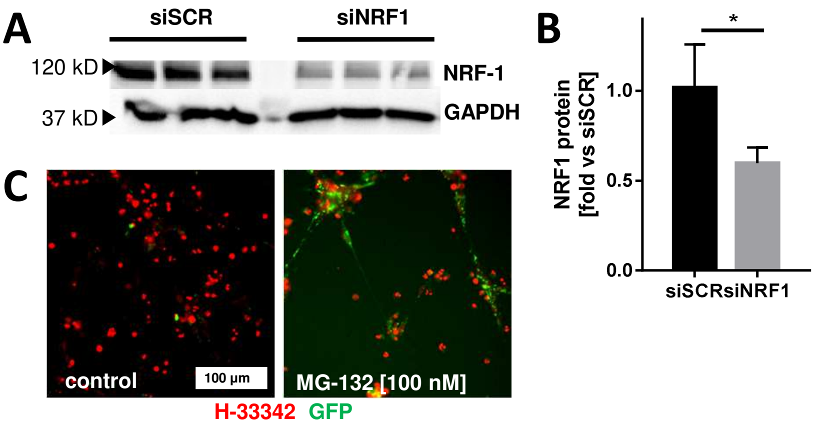

Fig. S14: Influence of NRF-1 abundance on survival after MG-132 exposure

A: LUHMES were transfected with siRNA against NRF-1 or scrambled siRNA at d2 of differentiation. Cells were cultured untill d6 of differentiation and then incubated with MG-132 [100 nM] for 7 h. After incubation, cells were lysed and analysed by Western blot using anti-NRF-1 and anti-GAPH antibodies. **B**: Densitometric quantification of NRF-1 protein levels following KD by siRNA. Differences between siSCR and siNRF1 treated samples were tested for significance, *: $p<0.05$ (*t*-test). **C:** LUHMES were transfected by electroporation with NRF-1 and GFP over-expression plasmids at d2 of differentiation. Cells were cultured until d6 of differentiation and then incubated with MG-132 [100 nM]. 18 h after MG-132 exposure, cells were stained with DNA stain H-33342 and double positive cells were counted by automated microscopy. Data quantification is displayed in main Fig. 5D+5F.

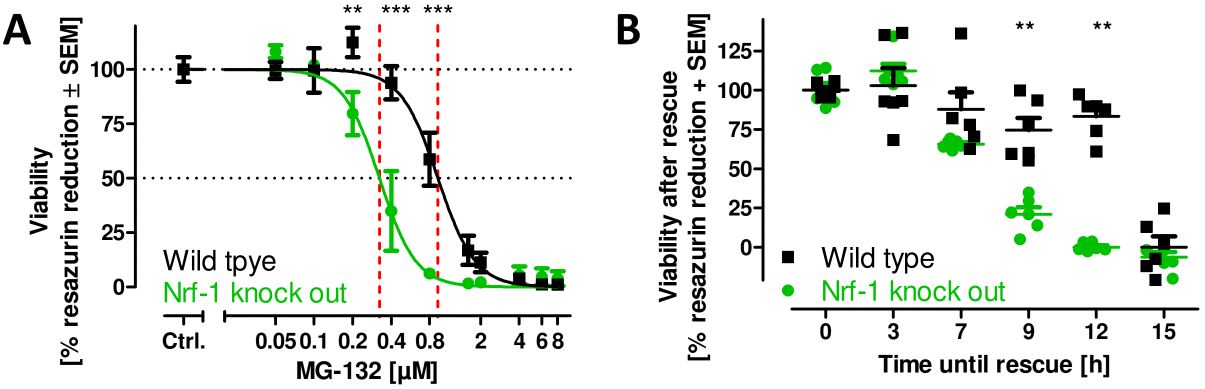

Fig. S15: Effect of Nrf-1 knockout on sensitivity to proteasomal inhibition by MG-132 and rescue by L-cysteine.

CRISPR/Cas technology was used to cut the Nrf-1 gene of HEK293 cells within exon 2 (at the position coding for tyrosine-50, in the PSSAY amino acid stretch). Single cell clone picking and testing yielded cells with a homozygous identical deletion of 22 base pairs, as confirmed by PCR strategies and by sequencing (cells here termed Nrf-1 knockout (ko)). The PSSAY sequence was modified to code for PSSTT, followed by a TGA stop codon (arising from the frame shift). For the experiment, the wild-type (wt) and Nrf-1 ko cells were cultured under identical standard conditions in 96-well dishes, and cell numbers were carefully controlled to be identical for testing.

A: Wild type and Nrf-1 knock-out (ko) cells were treated for 48 h with the indicated concentrations of MG-132. Cell viability was assessed by a resazurin reduction assay (and an LDH-release assay, not shown). Knock-out cells were found to be about 3-fold more sensitive (IC50(wt)= 0.9 µM; IC50(ko)= 0.3 µM) to MG-132 treatment than wt cells (red lines indicate IC50). Data are means ± SEM of three experiments run with entirely different start cultures. **B:** Cells were treated with MG-132 (2 µM) for the indicated times. Then, the drug was washed out and cysteine (1 mM) was added. Viability was assessed for all cultures (irrespective of washout time point) by resazurin reduction at 48 h after the start of the MG-132 treatment. Data were normalized to corresponding controls in which MG-132 was washed out directly after its addition (i.e. after 0 h). In addition to the individual data points, the means (large horizontal line) and the SEM (small horizontal line above) are depicted. For A+B, statistical significances were evaluated by two-way ANOVA for connected measures to compare different cells and treatments, followed by a Bonferroni post-hoc test to correct for multiple comparisons; ** $p < 0.01$, *** $p < 0.001$.

In brief, Nrf-1 ko cells were more susceptible to MG-132 treatment and a minimum exposure to MG-132 for 7 h was necessary to trigger cell damage, but under this condition, the separation between wt and ko was not significant. At later time points (9-12 h), wt cells were rescued to a significant extent (i.e. maintaining > 80% viability), while ko cells could not be rescued. At late time points (15 h and beyond), cell death had occurred in wt and ko cells and rescue was not possible anymore.

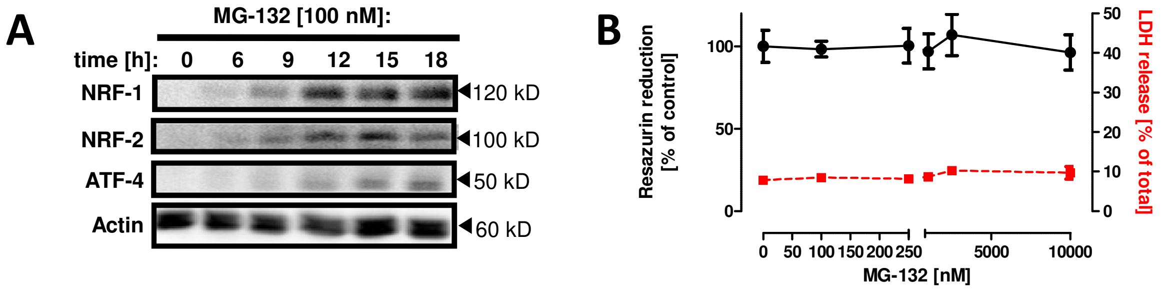

Fig. S16: Stress response in astrocytes triggered by MG-132

We observed in Figure 6A that the NRF-2 response was stronger in the co-culture compared to neuronal mono-cultures. This is consistent with published literature that astrocytes are the main cells in the brain showing an NRF-2 response (Vargas 2009), and in co-cultures we measured mainly astrocytic NRF-2. This was supported by the stress response observed in astrocytic mono-culture (Fig. S15A), and by resistance of astrocytes against MG-132 exposure (Fig. S15B).

A: To analyse the stress response following proteasome inhibition, astrocytic cells (mAGES) were treated with 100 nM MG-132 for the indicated time periods. After incubation, cells were lysed and analysed by Western blot with anti-NRF1, anti-ATF4, anti-NRF-2 and anti-beta-actin antibodies. The blots shown are representative for three experiments with similar results. **B:** mAges were treated for 24 h with indicated concentrations of MG-132. Viability was assessed by resazurin and LDH measurement. Data are means ± SD of three independent experiments.

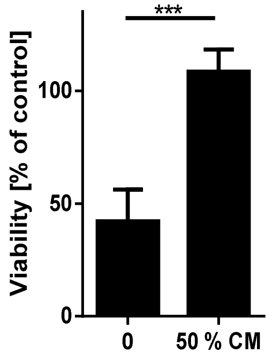

Fig. S17: Protection by astrocyte-conditioned medium against MPP⁺

LUHMES cells (d6) were cultured in fresh LUHMES culture medium only, or in astrocyte-conditioned medium from mAGES, diluted with fresh LUHMES culture medium (1:1). They were exposed to MPP⁺ [5 µM], and viability (resazurin reduction) was assessed 72 h after start of MPP⁺ exposure. Data are means ± SD from three experiments. The difference in viability was tested by Student's *t*-test and it was significant at the $p < 0.001$ level.

Fig. S18: Differential regulation of ATF-4 target genes in co- and mono-cultures following MPP⁺ exposure

To address differences in the stress responses following MPP⁺ exposure of co- and mono-cultures, gene expression was monitored after 36 h exposure to MPP⁺ [5 µM]. Relative regulation (to GAPDH) in co-and mono-culture is displayed in a x-y-graph. Data are means ± SEM of three independent experiments. To determine, whether co-cultures differed from mono-cultures, two statistical approaches were used (both based on mean regulation levels for each gene): a) two-way ANOVA indicated a influence of co-culture ($p < 0.01$). Post-hoc testing was not performed; b) regulation data were compared by a pair-wise Student's *t*-test (pairs were formed for each gene of the two treatments), $p = 0.013$.

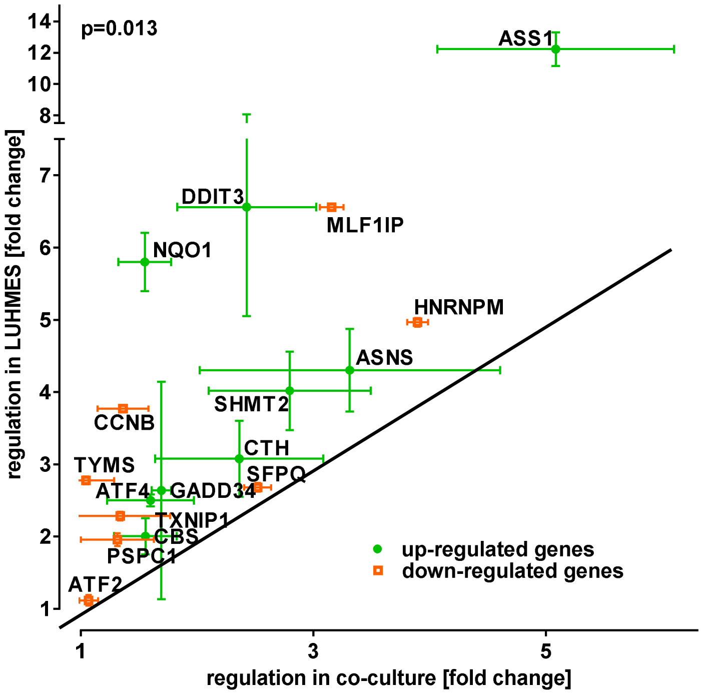

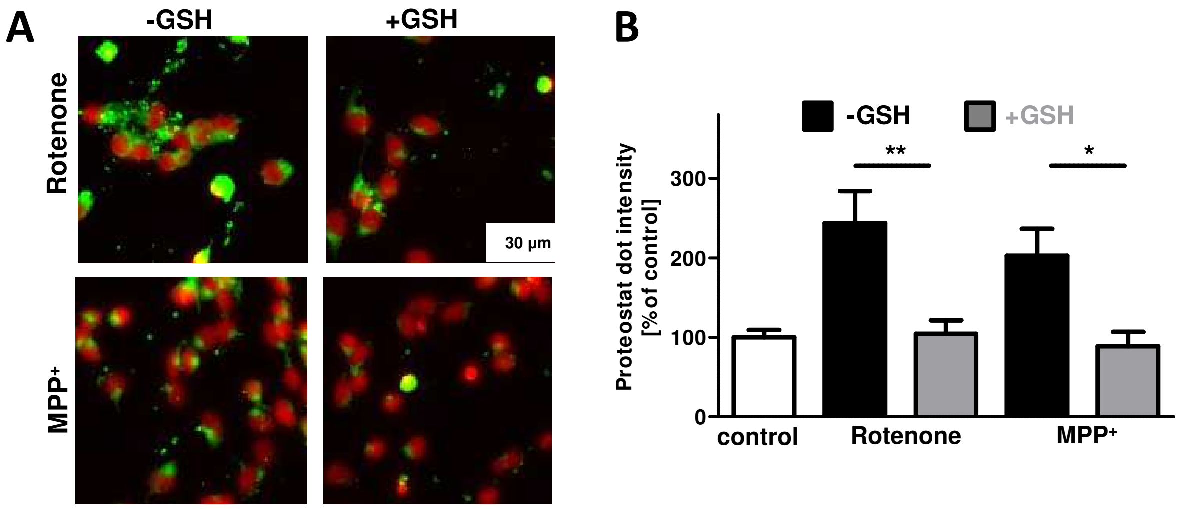

Fig. S19: Prevention of accumulation of protein aggregates in neurons

A: To test for proteasomal dysfunction, cells were treated with either rotenone [1 µM] or MPP⁺ [5 µM] for 24 h in presence or absence of GSH [1 mM]. Then, cells were fixed with paraformaldehyde (PFA), stained with Proteostat detection reagent (Enzo, Lausen, Switzerland) based on a dye binding to β-sheet protein aggregates, and analysed by automated microscopy. Aggregates are displayed in green, DNA (nuclei) are shown in red. **B:** Quantification of fluorescence intensity of the Proteostat reagent (measuring protein aggregates) in the samples described in A. Data are means ± SD of three independent experiments, **: $p<0.01$; *: $p<0.05$.

Antigen	Antibody (clone; supplier; catalog number)	Dilution	Blocking (5%)	Species
ATF4 (CREB-2)	Anti-ATF4, Rabbit mAb, (D4B8), Cell Signaling, (11815)	1:1000	BSA	rabbit
β- III-Tubulin	Mono β-Tubulin (TUJ1) purified, Covance (MMS-435P-250)	1:500	FCS	mouse
Cleaved Caspase 3	Cleaved Caspase- 3, Rabbit mAb, (5A1E), Cell Signaling (9664)	1:500	BSA	rabbit
DDIT4	REDD1 Polyclonal Antibody, Proteintech, (10638-1-AP)	1:1000	BSA	rabbit
Fodrin	α-Fodrin, mAb (AA6), Enzo Life Sciences (BML-FG60909	1:1000	BSA	mouse
GADD34	Anti-GADD34 (proteintech)	1:1000	BSA	mouse
GFAP	Monoclonal Anti-glial fibrillary acidic protein, Clone G-A-5, Sigma (G3893)	1:800	FCS	mouse
GAPDH	Anti-GAPDH (Sigma; Clone GAPDH-71.1)	1:5000	BSA	mouse
NOXA	NOXA Antibody, Mouse mAb (114C307.1), Novus, (NB600-1159)	1:1000	BSA	mouse
NRF1	TCF11/NRF1, Rabbit mAb, (D5B10), Cell Signaling (8052)	1:1000	BSA	rabbit
NRF2	NRF2, Rabbit mAb, (D1Z9C), Cell Signaling (12721)	1:1000	BSA	rabbit
PARP	PARP antibody, Cell Signaling (9542)	1:1000	BSA	rabbit
P-p70S6	Phospho-p70 S6 Kinase (Thr389), Cell Signaling (9205)	1:1000	BSA	rabbit
P-p38	Phospho-p38 MAPK (Thr180/182) antibody, Cell Signaling, (9211)	1:1000	BSA	rabbit
Ubiquitin	Ubiquitin, Mouse mAb, (P4D1), Cell Signaling (3936)	1:1000	BSA	mouse
anti-mouse (WB)	anti-mouse HRP antibody (Jackson Immuno Research)	1:2500	BSA	goat
anti-rabbit (WB)	anti-rabbit HRP antibody (GE Healthcare)	1:5000	BSA	goat
anti-mouse (ICC)	Alexa 488, IgG1 (γ1), goat anti mouse, Invitrogen, A21121	1:1000	FCS	goat
anti-mouse (ICC)	Alexa 555, IgG2a (γ2a), goat anti mouse, Invitrogen, A21137	1:1000	FCS	goat

Fig. S20: Antibodies used for Western blot or imunocytochemistry

Name	Forward sequence	Reverse Sequence
ASNS	GGGGCTTGGACTCCAGCTTG	GAGCCTGAATGCCTTCCTCA
ASS1	TGCTCCCTGGAGGATGCCTG	GTGTAGAGACCTGGAGGCGC
ATF2	AGAGCGAAATAGAGCAGCAG	CATGGCGGTTACAGGGCAAT
ATF4	GGCTGGCTGTGGATGGGTTG	CTCCTGGACTAGGGGGGCAA
CBS	TCCTGGGAATGGTGACGCTT	GTGCTGTGGTACTGGATCTG
CCNB	TGGATGTGCCCCTGCAGAAG	CAGTGACTTCCCGACCCAGT
CTH	TGGATGATGTGTATGGAGGTACAAACAGG	GCCTTCAATGTCAATCACCTTCTGGG
DDIT3/CHOP	ATGGCAGCTGAGTCATTGCC	TCCTCAGTCAGCCAAGCCAG
DDIT4	AGTCCCTGGACAGCAGCAAC	AACTGGCTAGGCATCAGCAG
GADD34	GCATCACCCAGGCCCAGGAG	AGACGAGCGGGAAGGTGTGG
GAPDH	CACCATCTTCCAGGAGCGAGATC	GCAGGAGGCATTGCTGATGATC
HNRNPM	TGGTGTGGTGGTCCGAGCAG	GGACGCTCAGGAGGGAAGAA
MLF1IP	TTTGTAAGGCAGCCATCGCC	CTGTGGCTCTAACCGAAGCA
NOXA	CAGTGCCAACTCAGCACATTG	CGCCCAACAGGAACACATTGA
NQO1	TGGAGTCGGACCTCTATGCCA	CTTGTGATATTCCAGTTCCCCCTGC
PSMA7	GCC GTC AAG AAG GGC TCG AC	CCA CCC GGG CCC TGT TGA TG
PSMB7	TCT CCA CTG GCC GTC TTC CC	AGC CAT TGC TGC CAA GGA GC
PSMC4	CAT CCC GCT GGT CAT CGG AC	ATG ATG CTG CTG TCG GCT TC
PSPC1	CAGCAGCGTGAGCAGGTTGA	CGCCGATGCTCCTCTTCATG
PSPH	CCC CGG CAT AAG GGA GCT GG	GCT GTT GGC TGC GTC TCA TC
SFPQ	TCAGGCAAATCTTTTGCGCC	CTCTCTTTGGCGCCTCATTT
SHMT2	CAACCTGGCACTGACTGCTC	GATGTCCGCGTGCTTGAAAG
SLC7A11	GCA GCG TGG GCA TGT CTC TG	CAC AGC AGT AGC TGC AGG GC
TXNIP1	CATGGCGTGGCAAGAGCCTT	CTCAGAGCTGGTTCGGCTGG
TYMS	CAGCTTCAGCGAGAACCCAG	ACCTCGGCATCCAGCCCAAC
VCP/P97	ATC AGC ATC CAG CCA TGC CC	CAC AGC ACG CAT CCC ACC AC

Fig. S21: Primers used for RT-qPCR

Supplementary References:

Astrocyte Differentiation of Human Pluripotent Stem Cells: New Tools for Neurological Disorder Research.
Chandrasekaran A, Avci HX, Leist M, Kobolak J, Dinnyes A.
Front Cell Neurosci. 2016 Sep 26;10:215. eCollection 2016.

Switching from astrocytic neuroprotection to neurodegeneration by cytokine stimulation.
Efremova L, Chovancova P, Adam M, Gutbier S, Schildknecht S, Leist M.
Arch Toxicol. 2017 Jan;91(1):231-246. doi: 10.1007/s00204-016-1702-2. Epub 2016 Apr 6.

The Nrf2-ARE cytoprotective pathway in astrocytes
Vargas MR, Johnson JA
Expert Rev Mol Med. 2009 Jun 3;11:e17. doi:10.1017/S1462399409001094
